# Decoding the Functional Proteome of *Vitis*: Past, Present, and Future

**DOI:** 10.3390/plants15091314

**Published:** 2026-04-24

**Authors:** Ivana Tomaz, Ana Jeromel, Darko Vončina, Ivanka Habuš Jerčić, Boris Lazarević, Iva Šikuten, Simona Hofer Geušić, Darko Preiner

**Affiliations:** 1 Department of Viticulture and Enology, Faculty of Agriculture, University of Zagreb, Svetošimunska 25, 10000 Zagreb, Croatia; amajdak@agr.hr (A.J.); shgeusic@agr.hr (S.H.G.); 2 Centre of Excellence for Biodiversity and Molecular Plant Breeding, Faculty of Agriculture, University of Zagreb, 10000 Zagreb, Croatia; dvoncina@agr.hr (D.V.); ihabus@agr.hr (I.H.J.); blazarevic@agr.hr (B.L.); 3 Department of Phytomedicine, Faculty of Agriculture, University of Zagreb, Svetošimunska 25, 10000 Zagreb, Croatia; 4 Department of Plant Breeding, Genetics and Biometrics, Faculty of Agriculture, University of Zagreb, Svetošimunska 25, 10000 Zagreb, Croatia; 5 Department of Plant Nutrition, Faculty of Agriculture, University of Zagreb, Svetošimunska 25, 10000 Zagreb, Croatia

**Keywords:** *Vitis* proteome, grapevine physiology, stress-responsive proteins, proteome remodeling, enological relevance

## Abstract

Proteomic research in the genus *Vitis* has progressed from early biochemical studies of soluble proteins to high-resolution, quantitative analyses encompassing all major organs and derived products. This review provides a comprehensive synthesis of advances in grapevine and wine proteomics. In leaves, studies have revealed extensive remodeling of photosynthetic, antioxidant, and defense pathways under biotic (e.g., *Plasmopara viticola*, *Erysiphe necator*, *Xylella fastidiosa*, *Candidatus Phytoplasma vitis*) and abiotic stresses (drought, salinity, heat, light). Bud proteomics elucidated hormonal regulation and mechanisms of dormancy release, while root studies identified nitrate-dependent metabolic shifts and adaptive protein networks. Cell culture models enabled controlled investigation of elicitor responses, stilbene biosynthesis, and temperature-induced proteome changes. In berries, proteomics clarified developmental transitions from fruit set to ripening, emphasizing proteins related to secondary metabolism, vacuolar transport, and stress tolerance. Comparative analyses across cultivars and environments identified biomarkers linked to aroma, color, and texture. The wine proteome revealed selective persistence of grape-derived proteins (e.g., thaumatin-like proteins, chitinases) and yeast peptides influencing stability and sensory properties, while Botrytis cinerea infection significantly alters this balance by degrading PR proteins and introducing fungal enzymes. Altogether, the Vitis proteome emerges as a dynamic, multifunctional system crucial for understanding plant adaptation, enological quality, and biomarker discovery.

## 1. Introduction

The genus *Vitis* is among the most economically important plant taxa. According to FAO data, global grape production in 2023 reached nearly 72.48 million tons, cultivated on 6.6 million hectares of vineyards [1]. The economic importance of *Vitis* species, particularly *Vitis vinifera*, underscores the necessity for advanced molecular approaches to enhance crop resilience and optimize yield and quality. In this context, proteomics has emerged as a powerful tool for understanding plant responses to environmental stresses and developmental processes, particularly following the availability of the grape genome sequence, which greatly facilitates protein identification [2,3].

Proteins are central functional molecules involved in virtually all biological processes, including metabolism, energy transfer, respiration, photosynthesis, cell division, DNA replication, gene expression regulation, molecular transport, cellular communication, and defense mechanisms [4]. In plants, proteomic analyses provide critical insights into molecular mechanisms that cannot be fully inferred from genomic or transcriptomic data alone [5]. Because protein abundance, localization, and activity are dynamically regulated and strongly influenced by environmental conditions, proteomics offers a direct link between genotype and phenotype.

In grapevine, proteomic studies have gained increasing importance due to both the biological complexity of the plant and its economic relevance. Beyond their roles in plant physiology, proteins significantly influence grape and wine quality. Despite their relatively low abundance, they contribute to key technological properties such as wine clarity, stability, foam formation, and potentially sensory characteristics [6,7]. In particular, pathogenesis-related proteins, including chitinases and thaumatin-like proteins, are among the most extensively studied due to their persistence during vinification and their role in haze formation [2].

The rapid development of proteomic technologies over the past two decades, particularly advances in mass spectrometry and bioinformatics, has substantially expanded our understanding of grapevine biology. These developments, together with the availability of genomic resources, have enabled high-resolution identification and characterization of proteins across different tissues, developmental stages, and environmental conditions [3]. Proteomic approaches have been widely applied to investigate plant responses to abiotic stresses such as drought, salinity, and temperature fluctuations, as well as interactions with pathogens. In parallel, they have provided valuable insights into grape berry development and the composition of the wine proteome.

Importantly, proteomic studies in grapevine have consistently shown that plant responses to environmental and developmental cues are governed not by individual proteins, but by coordinated changes in functional protein networks [2,3]. These networks involve proteins related to primary metabolism, redox homeostasis, stress signaling, and defense, which are dynamically reprogrammed depending on tissue type and external conditions. Such findings emphasize the need to consider the proteome as an integrated and highly context-dependent system. Indeed, similar functional groups of proteins frequently recur across different organs and stress conditions, reflecting conserved adaptive strategies at the proteome level [5,8].

Several review articles have addressed specific aspects of grape and wine proteomics, each contributing distinct perspectives to the field. Ferreira et al. [6] provided one of the earliest comprehensive overviews of wine proteins, focusing on their origin, structural characteristics, and technological relevance, particularly their role in haze formation and stability. Flamini and De Rosso [7] highlighted the application of mass spectrometry in the analysis of grape and wine proteins, emphasizing its importance for protein identification and characterization. Le Bourse et al. [8] offered a detailed methodological review of preparative and analytical approaches, critically evaluating chromatographic, electrophoretic, and mass spectrometry-based techniques. Giribaldi and Giuffrida [2] presented an integrative perspective on grapevine proteomics, linking protein dynamics to berry development, stress responses, and wine composition. Čarná et al. [5] expanded this framework by introducing proteomics and interactomics as complementary approaches for understanding complex biological systems and genotype–phenotype relationships. More recently, George and Haynes [3] focused on proteomic responses to abiotic stress, providing insights into the molecular mechanisms underlying plant adaptation to environmental challenges.

Despite these valuable contributions, existing reviews often remain focused on specific biological contexts, methodological aspects, or individual organs, and do not fully integrate the growing body of proteomic data across the grapevine system. Consequently, a comprehensive synthesis that connects proteomic responses across organs, biological processes, and environmental conditions is still lacking. In particular, the identification of recurring functional patterns and conserved protein responses across independent studies remains limited.

The present review addresses this gap by providing an updated and integrative overview of proteomic research in *Vitis*, structured around an organ-based framework encompassing leaves, roots, buds, berries, cultured cells, and derived products such as wine. This approach reflects the strong tissue specificity of proteomic responses and enables the interpretation of protein dynamics within biologically coherent systems, while integrating biotic, abiotic, and developmental influences within each organ-specific context. Rather than presenting a catalogue of individual findings, this review emphasizes recurrent functional trends and coordinated proteome remodeling, with the aim of identifying robust protein markers associated with stress tolerance, developmental processes, and enological traits.

## 2. Methods

This review is based on a comprehensive and structured survey of the scientific literature related to proteomic studies in the genus *Vitis*. Relevant publications were retrieved from major scientific databases, including Google Scholar, Web of Science, Scopus, and PubMed, covering the period from the 1950s to 2025. The literature search strategy combined proteomics-specific terms (e.g., “*Vitis* proteomics”, “grapevine proteome”, “wine proteomics”, “grape proteome”) with broader descriptors such as “proteins” and “protein analysis” and was further refined using organ-specific terminology. All identified studies relevant to proteomic analyses of grapevine organs (leaves, roots, buds, berries), cell cultures, and derived products such as wine were considered and included in the analysis. The collected data were qualitatively synthesized and systematically interpreted, and organized according to plant organs, biological processes, and stress conditions. Particular emphasis was placed on identifying recurring protein groups and conserved response patterns across independent studies, enabling the identification of consistent functional trends and potential biomarkers.

**Figure 1:**
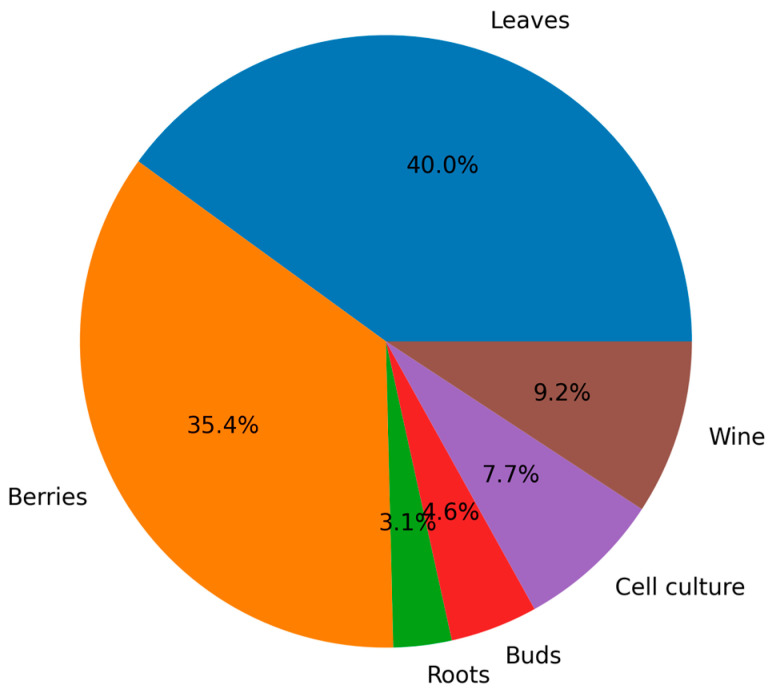
Distribution of proteomic studies across grapevine organs and derived products included in this review. Studies presented in Section 3 (Historical overview of protein research in *Vitis*) are not included in this distribution, as they were considered separately due to their historical and methodological scope.

The literature was primarily organized using an organ-based framework (leaves, roots, buds, berries, cultured cells, and derived products), rather than grouping studies by biotic or abiotic factors or developmental stages. This approach was adopted because proteomic responses in grapevine are strongly tissue-specific, with each organ exhibiting distinct metabolic functions, physiological roles, and stress-response mechanisms. Organ-based structuring allows for a clearer interpretation of proteome dynamics within biologically coherent systems, while still enabling the integration of biotic, abiotic, and developmental influences within each organ-specific context. The distribution of available studies across different organs was uneven, reflecting the current research landscape. The majority of studies focused on leaves (*n* = 52) and berries (*n* = 46), which represent the most extensively investigated systems in *Vitis* proteomics. In contrast, substantially fewer studies addressed roots (*n* = 4) and buds (*n* = 6), while intermediate coverage was observed for cell cultures (*n* = 10) and derived products such as wine (*n* = 12) as illustrated in Figure 1. These counts do not include studies discussed in Section 3 (Historical overview of protein research in *Vitis*) which were considered separately due to their historical and methodological scope. This disparity was considered when structuring the review and determining the level of detail across sections.

For the construction of summary figures, differentially expressed proteins (DEPs) reported in individual studies were extracted and assigned to functional categories based on their annotated biological roles (e.g., photosynthesis, stress response, defense, metabolism, etc.). Within each dataset, the number of proteins in each category was expressed as a proportion of the total number of reported DEPs and normalized to 100%. Accordingly, the presented values reflect the relative distribution of functional categories rather than protein abundance. Because the analysis is based on heterogeneous literature-derived datasets differing in experimental design, analytical platforms, and reporting criteria, no formal statistical meta-analysis or cross-study quantitative comparison was performed. This approach allows the integration of diverse proteomic datasets while preserving biologically meaningful patterns across studies.

## 3. Historical Overview of Protein Research in *Vitis*

The history of protein analysis dates back to the early 20th century; however, the development of two-dimensional gel electrophoresis (2-DE) in 1975 marked the beginning of large-scale protein studies. Further progress was achieved with the introduction of immobilized pH gradient (IPG) strips in 1982 and the establishment of “soft” ionization methods such as electrospray ionization (ESI) and matrix-assisted laser desorption/ionization (MALDI), both coupled to mass spectrometry (MS). These advances shaped proteomics into a modern scientific discipline and enabled high-resolution protein identification and characterization. The term “proteomics” was first introduced by Mark Wilkins and refers to the study of the complete set of proteins expressed by a genome under specific conditions. Unlike genomic data, the proteome reflects both genetic programming and environmental influences, as proteins are dynamic entities subject to extensive regulation and post-translational modifications (PTMs). Consequently, proteomic analysis provides essential information on protein expression, modification, localization, interactions, and function, offering a more accurate representation of the cellular phenotype than transcriptomic data alone [9,10,11,12,13].

In plant systems, proteomic analysis presents additional challenges due to the structural and biochemical complexity of plant tissues. The presence of rigid cell walls, plastids, and vacuoles rich in secondary metabolites can interfere with protein extraction and analysis, while proteins represent a relatively small proportion of total cellular mass. Despite these limitations, plant proteomics has advanced considerably and now encompasses subcellular, developmental, and stress-related proteomics, as well as studies of PTMs and protein–protein interactions [11]. These methodological developments have laid the foundation for the application of proteomic approaches in complex plant systems, including grapevine.

### 3.1. Historical Development of Analytical Methods

At the beginning of the 20th century, it was already recognized that certain grape proteins could persist through fermentation and cause haze in wines. Systematic research began in the 1950s with the group of Koch, who analyzed proteins in free-run juice. Because these proteins were soluble in must, they were referred to as “soluble proteins.” Using paper electrophoresis, the group estimated isoelectric points (pI) between 3.3 and 3.7 and separated proteins into five fractions, with banding patterns depending on grapevine variety [14].

In the 1960s, Berg and colleagues introduced polyacrylamide gel electrophoresis (PAGE), resolving grape juice proteins into 16 bands with molecular weights between 18 and 23 kDa [15,16]. These methodological advances established the foundation for more detailed protein separation and characterization.

During the 1970s, Anelli’s group further investigated soluble proteins, reporting eight fractions that differed by pI (2.5–8.7), with acidic proteins predominating and comprising approximately 75% of total protein content [17,18]. At the same time, starch gel electrophoresis was applied for isozyme analysis across multiple cultivars, enabling varietal differentiation based on enzyme banding patterns [19].

In the 1980s, analytical resolution improved further, allowing soluble proteins to be resolved into dozens of fractions [20,21,22], and enabling comparative analyses between *Vitis vinifera* and *Muscadinia* species based on molecular weight and isoelectric point differences [23]. Electrophoretic and isozyme profiling approaches were also explored for detecting viral infections and for studying inheritance patterns relevant to breeding programs [24,25].

A major methodological breakthrough occurred in the early 1990s with the introduction of two-dimensional electrophoresis (2-DE), which enabled high-resolution separation of proteins based on both isoelectric point and molecular weight. This approach facilitated detailed characterization of proteins from musts [26], berries [27], and seeds [28,29], and provided a robust analytical platform for varietal differentiation and statistical discrimination of grape musts [30,31,32].

The integration of mass spectrometry–based approaches in the 1990s further expanded protein identification capabilities. A milestone was achieved in 1996, when Waters et al. [33] identified pathogenesis-related (PR) proteins as the primary contributors to wine haze, marking the transition from descriptive protein profiling to molecular-level characterization of grape proteins.

### 3.2. Key Biological Conclusions from Early Protein Studies

In parallel with methodological developments, early protein studies provided important insights into the composition, variability, and biological roles of grapevine proteins. Initial investigations demonstrated that protein profiles varied among grapevine varieties, indicating their potential use as biochemical markers [14,19].

During the 1960s, Harada [34] reported a cytokinin-binding protein in grape berries, representing one of the earliest attempts to assign functional significance to individual grape proteins. In the following decades, attention expanded toward enzymes with central roles in grapevine metabolism, including phosphoenolpyruvate carboxylase, peroxidases (PERs), phenylalanine ammonia-lyase (PAL), alcohol dehydrogenase (ADH), and polyphenol oxidase (PPO) [34,35,36,37,38,39,40,41,42]. These findings highlighted the involvement of proteins in primary metabolism and stress-related processes.

Protein profiling approaches were also applied to specific tissues. Analyses of pollen wall proteins revealed clone-specific banding patterns that were unaffected by rootstock or vineyard location [43,44]. Studies on seed storage proteins identified large protein assemblies composed of covalently and non-covalently linked subunits [45]. In addition, protein expression profiling was used to monitor somatic embryogenesis, where lipid transfer proteins (LTPs) and peroxidases were identified as key components of embryogenic development [46,47,48].

Isoenzyme and protein profiling approaches further demonstrated their utility in varietal identification and breeding. Protein fraction profiles enabled differentiation of grape varieties in must and wine [30], while native electrophoretic profiling reliably discriminated *V. vinifera* cultivars [31]. Statistical analysis of electrophoretic patterns was successfully applied to distinguish grape musts from different varieties [32], confirming the value of protein-based markers in grapevine classification.

A major biological breakthrough occurred with the identification of pathogenesis-related (PR) proteins as key components of grape and wine proteomes. Waters et al. [33] demonstrated that chitinases and thaumatin-like proteins are the primary contributors to wine haze, establishing the first direct link between grape proteins and enological stability. Subsequent studies confirmed the presence of multiple chitinase isoforms in grapevine tissues [49], their expression in berries [50], and their abundance in must [48,51]. Parallel work identified and characterized thaumatin-like proteins (TLPs), whose accumulation coincided with sugar accumulation and berry softening during ripening [52,53].

Further mass spectrometry–based analyses expanded the classification of grape proteins into PR families, including PR-3 chitinases, PR-4 proteins, and PR-5 TLPs [54]. Later studies revealed that environmental conditions and developmental stage strongly influence the abundance and diversity of PR proteins, which typically increase during ripening while their diversity decreases [55,56]. These findings demonstrated that the grape proteome is dynamic and tightly regulated in response to both physiological and environmental factors.

### 3.3. Transition Toward Integrative Proteomics

**Figure 2:**
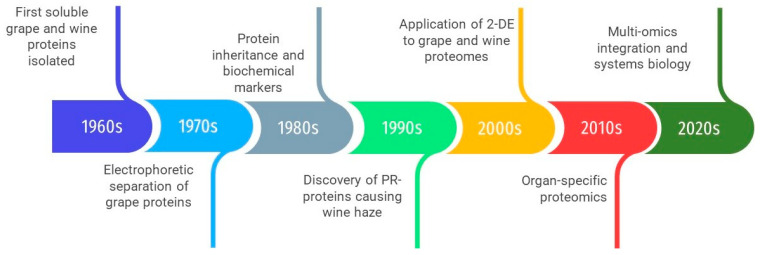
Timeline of key milestones in protein and proteomics research in *Vitis*.

The historical progression of protein research in *Vitis* mirrors the broader evolution of analytical biochemistry and proteomics. As outlined in Figure 2, the field advanced from early isolation of soluble proteins and electrophoretic separation to the identification of biochemical markers and functionally relevant protein groups. The introduction of two-dimensional electrophoresis and mass spectrometry marked the beginning of large-scale protein analysis, while subsequent developments enabled quantitative and systems-level approaches.

This methodological and conceptual evolution laid the foundation for modern grapevine proteomics. The following sections build on this continuum, examining the proteomic landscapes of distinct organs—leaves, buds, roots, berries, and cultured cells—and their roles in mediating stress responses, developmental transitions, and enological traits.

## 4. Leaf Proteomics

Proteomic studies of grapevine leaves in the absence of defined stress conditions have provided fundamental insights into the molecular composition of this organ. They highlight not only the dominance of photosynthetic proteins but also the presence of metabolic, signaling, and defense-related proteins that contribute to the dual role of leaves as both photosynthetic and protective tissues.

In *Vitis vinifera*, proteomic analyses have revealed a complex protein composition that integrates photosynthetic activity with structural and defense-related functions. Proteomic profiling of xylem sap in *V. vinifera* Chardonnay identified seven abundant proteins, including subtilisin-like proteases, α-L-arabinofuranosidase, β-D-xylosidase, glycine-rich proteins, peroxidases, and chitinases [57]. These proteins are primarily associated with cell wall modification, lignification, and constitutive defense, indicating that structural and protective processes are closely linked to leaf metabolism.

Secreted and apoplastic proteins represent another important fraction of the *V. vinifera* leaf proteome. Figueiredo et al. identified a diverse array of proteins in the apoplast, including PR-2 β-1,3-glucanases, PR-3/4 chitinases, PR-5 thaumatin-like proteins, class III peroxidases, expansins, pectinesterases, and protease inhibitors, highlighting a constitutive defense signature at the leaf surface [58]. Complementary large-scale mapping identified 177 proteins, revealing a dominance of PR proteins, redox-related enzymes, and cell wall–modifying proteins such as expansins and pectinesterases [59].

Comparative proteomic analyses across *Vitis* species provide key insights into the organization of the leaf proteome. A study including *V. vinifera*, *V. rotundifolia*, and Florida hybrid bunch cultivars resolved over 255 proteins, with 54 being differentially expressed [60]. In this analysis, *V. vinifera* leaves were characterized by enhanced accumulation of photosynthesis-related proteins, including ribulose-1,5-bisphosphate carboxylase/oxygenase (RuBisCO) isoforms, chlorophyll a/b binding proteins, oxygen-evolving enhancer proteins, and key enzymes of the Calvin cycle such as glyceraldehyde-3-phosphate dehydrogenase and phosphoglycerate kinase. In contrast, muscadine (*V. rotundifolia*) leaves exhibited higher abundance of defense-related proteins such as PR-10 proteins, peroxidases, chitinases, β-1,3-glucanases, and thaumatin-like proteins, while Florida hybrids showed an intermediate proteomic profile enriched in proteins associated with protein trafficking, molecular chaperones (HSP70 family), and enzymes of secondary metabolism. These interspecific differences are further supported by detailed proteomic analyses of muscadine grape (*V. rotundifolia*) leaves, where more than 250 proteins were resolved using 2-DE, including major photosynthetic proteins such as RuBisCO subunits, oxygen-evolving enhancer proteins, chlorophyll a/b binding proteins, and ATP synthase subunits [61]. In addition, enzymes such as glutamine synthetase and glyoxysomal malate dehydrogenase were abundant, reflecting active nitrogen and carbon metabolism. Notably, ribonucleoproteins and several pathogenesis-related (PR) proteins were also identified, indicating that constitutive basal defense mechanisms are present even under non-stressed conditions.

Together, these studies demonstrate that the grapevine leaf proteome integrates two major functional layers: photosynthetic and primary metabolic processes, and defense- and stress-related components. Comparative analyses suggest that these functional layers are differentially balanced across *Vitis* species, reflecting distinct evolutionary strategies that integrate productivity with inherent stress resistance.

### 4.1. Leaf Proteomics Under Biotic Stress

#### 4.1.1. Proteome Changes Under *Plasmopara viticola* Infection

Downy mildew, caused by the obligate biotrophic oomycete *Plasmopara viticola*, is among the most devastating foliar diseases of grapevine. It is an obligate biotrophic oomycete that colonizes intercellular spaces and forms haustoria within host cells, allowing nutrient acquisition without immediate tissue necrosis. Proteomic investigations have substantially contributed to unraveling the molecular basis of host–pathogen interactions, highlighting differences between compatible and incompatible responses, resistant and susceptible genotypes, as well as elicitor-induced resistance.

Apoplastic proteomics at the earliest stages of infection (6 h post-infection; hpi) demonstrated a rapid accumulation of cell wall–modifying enzymes (expansins, β-1,3-glucanases, pectinesterases) and ROS-related proteins (peroxidases, superoxide dismutase, ascorbate peroxidase), reflecting an oxidative burst and reinforcement of the cell wall [62,63]. These studies used LC–MS/MS shotgun proteomics to profile both host and pathogen proteins. In parallel, pathogen-secreted cell wall–degrading enzymes and effector proteins were identified [64], highlighting the apoplast as a critical site of reciprocal signaling and metabolic reprogramming.

In susceptible cultivars such as Pinot Noir, infection caused a pronounced downregulation of photosynthetic proteins including RuBisCO subunits, chlorophyll a/b binding proteins, oxygen-evolving enhancer proteins, and ATP synthase subunits [65,66]. These findings were obtained using 2-DE gels coupled with MALDI-TOF/TOF or LC–MS/MS, demonstrating that the decline in photosynthetic activity and ATP synthesis compromised energy availability for defense. Such suppression likely reflects pathogen-induced reallocation of host resources toward defense rather than growth. In parallel, induction of PR-10 proteins was observed, but the delayed timing led to an ineffective response [65]. In contrast, resistant cultivars such as Regent exhibited an early induction of ROS-detoxifying enzymes (superoxide dismutase, ascorbate peroxidase, glutathione S-transferase, catalase), lipid metabolism enzymes (lipoxygenases, 12-oxophytodienoate reductase), and jasmonate-related proteins [65,67]. These results were obtained through comparative 2-DE proteomics and LC–MS/MS, highlighting robust metabolic shifts in resistant genotypes. The accumulation of heat shock proteins (HSP70, HSP90) further indicated enhanced protein stability and stress signaling [67].

Grapevine genotypes harboring pyramided resistance loci *Rpv1* and *Rpv3* displayed distinct proteomic reprogramming [68]. Using a label-free quantitative LC–MS/MS approach, Nascimento-Gavioli et al. identified more than 800 proteins and revealed downregulation of proteins associated with hypersensitive cell death, together with upregulation of primary metabolic enzymes (glyceraldehyde-3-phosphate dehydrogenase, malate dehydrogenase, citrate synthase). By sustaining central carbon metabolism while limiting cell death, these genotypes maintain energy homeostasis and achieve durable resistance.

Treatment with *Trichoderma harzianum* T39 led to the accumulation of phenylalanine ammonia-lyase, class III peroxidases, and other enzymes of the phenylpropanoid pathway, enhancing lignin and stilbene biosynthesis [69]. This was shown using 2-DE proteomics coupled with MALDI-TOF/TOF. Similarly, sulfated laminarin (PS3) elicitation triggered induction of 12-oxophytodienoate reductase (OPR-like), together with enzymes from amino acid (aspartate aminotransferase, glutamine synthetase) and carbohydrate metabolism (fructose-bisphosphate aldolase, enolase, pyruvate kinase) [70]. Lemaitre-Guillier et al. employed 2-DE/MS-based proteomics to demonstrate that protective elicitors reprogram both primary and secondary metabolism, whereas non-protective elicitors mainly induced PR proteins without major metabolic changes. Proteomic approaches also identified two candidate glycoproteins potentially involved in the abnormal stomatal deregulation seen in infected leaves. Guillier et al. used a glycoprotein enrichment strategy followed by LC–MS/MS to show that instead of closing as part of the defense response, stomata remained open, facilitating pathogen entry and sporulation [71].

**Figure 3:**
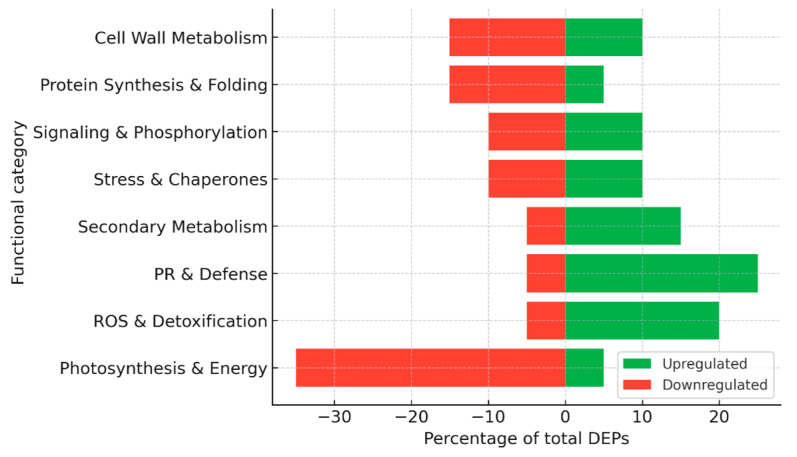
Distribution of differentially expressed proteins (DEPs) across functional categories in grapevine leaves infected with *Plasmopara viticola*. Proteins were classified according to their biological functions. Values represent the proportion of upregulated and downregulated proteins within each category, normalized to 100% for the dataset. These values reflect the distribution of functional categories rather than absolute protein abundance. DEPs were extracted from literature sources, and no cross-study statistical analysis was performed due to dataset heterogeneity.

Collectively, proteomic studies have revealed several hundred differentially expressed proteins during grapevine–*P. viticola* interactions (Figure 2), with variations depending on the genotype, time point, and methodological approach, including 2-DE, MALDI-TOF/TOF, LC–MS/MS, label-free quantification, and glycoprotein enrichment. A consistent trend in susceptible cultivars is the suppression of photosynthesis and primary energy metabolism, which compromises the energy supply needed for defense. In contrast, resistant cultivars typically display a rapid induction of ROS-detoxifying enzymes, heat shock proteins, and jasmonate-related enzymes, reflecting an enhanced capacity to stabilize metabolism and signal stress responses. Elicitor and biocontrol treatments promote the activation of the phenylpropanoid pathway and the accumulation of stilbenes, thereby strengthening cell walls and secondary defenses. In addition, specific pathogen effectors and glycoproteins have been identified that play key roles in early infection events and stomatal regulation. The overall distribution of functional categories among up- and down-regulated proteins is summarized in Figure 3.

#### 4.1.2. Proteome Changes Under *Erysiphe necator* Infection

Powdery mildew, caused by the obligate biotrophic fungus *Erysiphe necator* (syn. *Uncinula necator*), is one of the most destructive foliar diseases of grapevine. *E. necator* is an obligate biotrophic fungus that develops on the leaf surface while extracting nutrients through haustoria. Proteomic studies have provided key insights into how susceptible cultivars are reprogrammed during infection and how mineral nutrition, such as manganese supply, can enhance resistance.

In the susceptible cultivar Cabernet Sauvignon, iTRAQ-based quantitative proteomics identified 63 proteins with significantly altered abundance during infection (24–72 hpi). The most consistent changes occurred in photosynthesis-related proteins. Large and small subunits of ribulose-1,5-bisphosphate carboxylase/oxygenase (RuBisCO), chlorophyll a/b binding proteins, oxygen-evolving enhancer proteins, ferredoxin-NADP reductase, and multiple ATP synthase subunits were strongly downregulated, reflecting a marked suppression of energy production. Because host tissues remain largely intact, these changes reflect metabolic reprogramming rather than structural damage. At the same time, proteins related to defense and stress were induced. These included PR-10 family proteins, beta-1,3-glucanase, and thaumatin-like proteins. Redox-related enzymes such as ascorbate peroxidase, catalase, and glutathione-S-transferase showed dynamic changes in abundance, indicating an oxidative burst accompanied by an attempt to restore redox homeostasis. Secondary metabolism was also impacted, with phenylalanine ammonia-lyase (PAL) and chalcone synthase showing altered expression, suggesting reprogramming of the phenylpropanoid pathway [72].

In contrast, leaves pretreated with manganese (Mn) exhibited a more balanced proteomic response when infected with *E. necator*. 2D-DIGE analysis identified 74 differentially expressed proteins, many overlapping functionally with those from the susceptible interaction. However, in Mn-treated leaves, suppression of photosynthetic proteins such as RuBisCO, oxygen-evolving enhancer protein, and chlorophyll a/b binding proteins was less pronounced, allowing photosynthetic activity and ATP production to be maintained. In addition, Mn treatment enhanced the abundance of several defense- and signaling-related proteins. These included peroxidases, PR-5 thaumatin-like proteins, and enzymes of the phenylpropanoid and lignin biosynthesis pathways such as caffeoyl-CoA O-methyltransferase. Enzymes of glycolysis and the TCA cycle, including glyceraldehyde-3-phosphate dehydrogenase and malate dehydrogenase, were also maintained or upregulated, supporting metabolic energy requirements during defense activation. Heat shock proteins (HSP70, HSP90) and molecular chaperones were more abundant in Mn-treated plants, suggesting a role in protein stabilization and enhanced stress tolerance [73].

**Figure 4:**
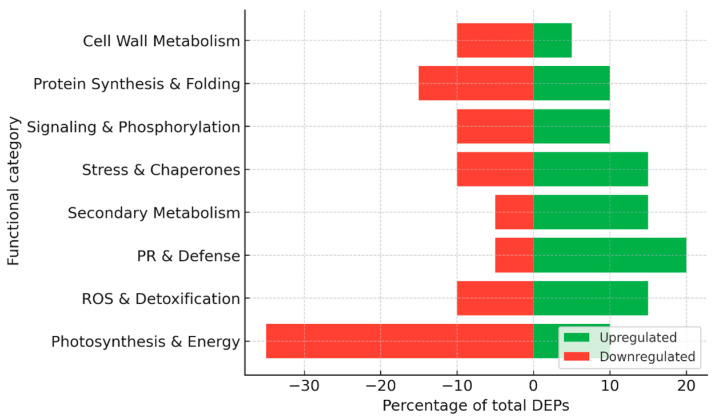
Distribution of differentially expressed proteins (DEPs) across functional categories in grapevine leaves infected with *Erysiphe necator*. Proteins were classified according to their biological functions. Values represent the proportion of upregulated and downregulated proteins within each category, normalized to 100% for the dataset. These values reflect the distribution of functional categories rather than absolute protein abundance. DEPs were extracted from literature sources, and no cross-study statistical analysis was performed due to dataset heterogeneity.

Together, these studies highlight a dual strategy in grapevine–*E. necator* interactions: in susceptible cultivars, the pathogen causes a sharp downregulation of photosynthetic machinery and energy metabolism, while inducing delayed defense proteins, leading to ineffective resistance (Figure 4). By contrast, Mn treatment promotes a proteomic state in which energy metabolism is sustained and defense responses, including PR proteins, phenylpropanoid enzymes, and redox regulators, are more robustly induced. This metabolic stabilization, combined with enhanced stress protection, underlies the increased resistance observed in Mn-supplemented grapevine leaves.

#### 4.1.3. Proteome Changes Under Esca Disease Complex Infection and Apoplexy

Esca disease complex is a chronic grapevine trunk disease characterized by tiger-stripe leaf symptoms and internal wood necrosis, caused by fungal genera such as *Phaeomoniella*, *Phaeoacremonium*, and *Fomitiporia*, while apoplexy represents its acute manifestation, often resulting in sudden vine collapse. Esca is a grapevine trunk disease caused by wood-inhabiting fungi, and leaf symptoms arise primarily from systemic effects of vascular dysfunction and fungal toxins rather than direct leaf infection. Proteomic studies have provided valuable insight into the molecular processes underlying these contrasting syndromes by examining both green shoots and woody tissues.

In green stems of Chardonnay, proteomic analysis revealed extensive changes in proteins linked to primary metabolism, stress tolerance, and defense. Components of energy metabolism, including ATP synthase subunits, malate dehydrogenase, and oxygen-evolving enhancer proteins, were often downregulated, suggesting impaired energy production. At the same time, stress-related proteins such as HSP70, small heat shock proteins, and GroEL-like chaperonins accumulated, reflecting the activation of protein stability and repair mechanisms. Defense-associated proteins, notably thaumatin-like proteins of the PR-5 family, peroxidases, and glutathione *S*-transferases, also increased in abundance. Interestingly, these alterations were not limited to symptomatic stems; even asymptomatic tissues displayed similar proteomic changes, indicating that the molecular impact of esca proper begins well before visible signs of the disease become apparent [74].

Proteomic analysis of woody tissues provided complementary evidence of how *Esca proper* and apoplexy differ in their molecular basis. In vines affected by esca proper, proteins associated with photosynthesis and respiration, such as RuBisCO subunits, ATP synthase, and oxygen-evolving enhancer proteins, were consistently reduced, while antioxidant and defense-related proteins, including ascorbate peroxidase, superoxide dismutase, glutathione S-transferases, peroxidases, and polyphenol oxidases, were induced. This pattern reflects a chronic rebalancing of the proteome in which suppressed energy metabolism is partially compensated by enhanced detoxification of reactive oxygen species and activation of defense pathways. In contrast, apoplexy was marked by a more dramatic proteomic deregulation. The depletion of proteins involved in energy metabolism was more severe, and the massive accumulation of oxidative stress markers and defense-related proteins pointed to an acute metabolic breakdown that corresponds with the sudden collapse of vines [75].

**Figure 5:**
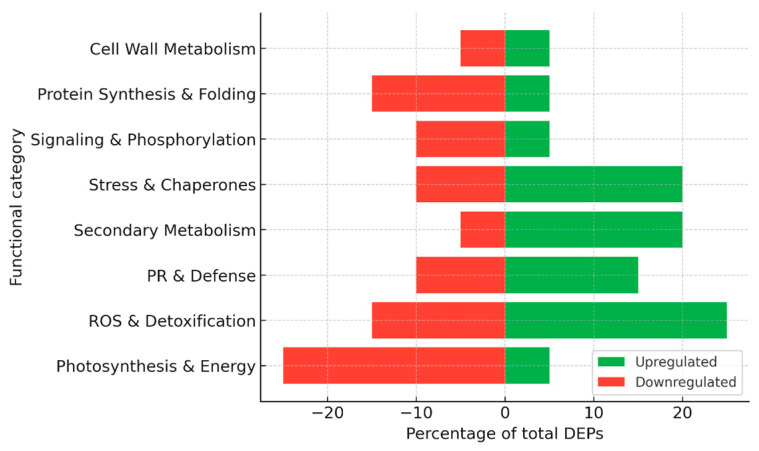
Distribution of differentially expressed proteins (DEPs) across functional categories in grapevine wood and green stems affected by Esca proper. Proteins were classified according to their biological functions. Values represent the proportion of upregulated and downregulated proteins within each category, normalized to 100% for the dataset. These values reflect the distribution of functional categories rather than absolute protein abundance. DEPs were extracted from literature sources, and no cross-study statistical analysis was performed due to dataset heterogeneity.

Taken together, these studies indicate that *Esca proper* is associated with progressive and chronic proteomic adjustments, where reduced energy metabolism is counterbalanced by sustained induction of stress and defense proteins, allowing infected vines to survive for years despite ongoing damage (Figure 5). Apoplexy, on the other hand, represents the acute endpoint of these processes, in which the balance between metabolism and stress tolerance collapses, leading to rapid vine death.

#### 4.1.4. Proteome Changes Under *Flavescence dorée* Infection

Flavescence dorée (FD) is a severe grapevine yellows disease caused by the phloem-restricted phytoplasma ‘*Candidatus Phytoplasma vitis’*, a quarantine pathogen in many viticultural regions. Phytoplasmas are wall-less bacteria that are strictly confined to the plant phloem. FD is considered the most important phytoplasma disease of grapevine. FD should be distinguished from other grapevine yellows diseases such as Bois noir, caused by ‘*Candidatus Phytoplasma solani’*, which differs in epidemiology and vector biology. The pathogen is transmitted in a persistent propagative manner by the leafhopper *Scaphoideus titanus* and colonizes the phloem, disrupting assimilate transport and source–sink relationships. As a consequence, infected vines exhibit severe alterations in photosynthesis, carbohydrate metabolism, and overall plant vitality. Proteomic studies by Margaria and Palmano have provided key insights into the molecular basis of grapevine responses to FD infection by comparing two cultivars with contrasting susceptibility: the highly sensitive Nebbiolo and the more tolerant Barbera, which can exhibit partial recovery in subsequent years.

In Nebbiolo, FD infection induces profound proteome reorganization, as demonstrated by Margaria and Palmano [76]. Using two-dimensional electrophoresis and LC–MS/MS, they identified a broad suppression of proteins related to photosynthesis and energy metabolism, including the large and small subunits of ribulose-1,5-bisphosphate carboxylase/oxygenase (RuBisCO), chlorophyll a/b binding proteins, oxygen-evolving enhancer proteins, ATP synthase subunits, and ferredoxin-NADP reductase. Enzymes involved in carbon fixation and glycolysis, such as fructose-bisphosphate aldolase, enolase, glyceraldehyde-3-phosphate dehydrogenase, and phosphoglycerate kinase, were also reduced in abundance. This global metabolic depression reflects a severe impairment of photosynthetic capacity and carbohydrate metabolism. Because FD phytoplasma is confined to the phloem, many of these metabolic disturbances likely result from impaired assimilate transport rather than direct tissue destruction. Concurrently, defense and stress-associated proteins accumulated, including thaumatin-like proteins (PR-5), osmotin, PR-10 proteins, and several enzymes of the antioxidant system, such as peroxidases, superoxide dismutase (SOD), and glutathione S-transferase (GST). Heat shock proteins (HSP70 and HSP90) and molecular chaperones were also strongly induced, suggesting the activation of protein protection and folding mechanisms. However, despite this intense stress response, the activation of defense pathways in Nebbiolo did not prevent disease progression. The persistence of FD symptoms and the lack of metabolic recovery indicate that the proteomic changes in Nebbiolo represent an unsuccessful compensatory attempt to cope with the infection [76].

In contrast, Barbera exhibits a more balanced and dynamic response to FD, as shown by Margaria and Palmano [77]. Proteomic and phosphoproteomic analyses revealed that, during the first year of infection, Barbera leaves displayed similar early alterations to those observed in Nebbiolo: strong downregulation of photosynthetic proteins (RuBisCO, chlorophyll a/b binding proteins, oxygen-evolving enhancer proteins) and enzymes of the Calvin cycle and glycolysis, combined with the induction of PR proteins and antioxidant enzymes. However, in the second year of infection—when plants began to exhibit recovery—several key photosynthetic and metabolic proteins, such as RuBisCO, ATP synthase, and enzymes of the tricarboxylic acid cycle (aconitase, malate dehydrogenase, isocitrate dehydrogenase), partially regained their normal abundance. This metabolic recovery was accompanied by a stabilization of redox homeostasis, as reflected by moderated expression of GST, peroxidases, and catalase, and by a reduction in the accumulation of stress-related chaperones. This recovery phenomenon, previously reported in FD-infected vines, suggests activation of long-term tolerance mechanisms rather than complete elimination of the pathogen. Indeed, FD phytoplasma often persists in recovered plants, indicating a state of functional tolerance rather than pathogen clearance. Interestingly, phosphoproteomic profiling demonstrated that recovery was not merely a reversal of infection-induced changes but involved active regulatory adjustments at the post-translational level. Proteins involved in signal transduction, cytoskeleton organization, and energy metabolism exhibited altered phosphorylation patterns, suggesting the activation of specific signaling pathways associated with tolerance and physiological re-equilibration [77].

**Figure 6:**
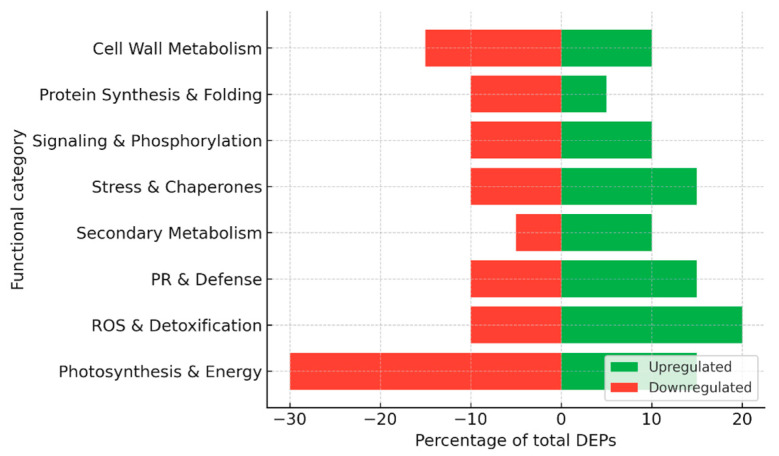
Distribution of differentially expressed proteins (DEPs) across functional categories in grapevine leaves infected with Flavescence dorée phytoplasma. Proteins were classified according to their biological functions. Values represent the proportion of upregulated and downregulated proteins within each category, normalized to 100% for the dataset. These values reflect the distribution of functional categories rather than absolute protein abundance. DEPs were extracted from literature sources, and no cross-study statistical analysis was performed due to dataset heterogeneity.

Together, these two studies highlight fundamentally different proteomic strategies underlying susceptibility and tolerance to FD phytoplasma (Figure 6). In the highly susceptible Nebbiolo, FD triggers a collapse of photosynthetic and metabolic networks, while the activation of defense and stress proteins remains insufficient to restore physiological balance. In contrast, Barbera displays proteome and phosphoproteome plasticity that allows metabolic recovery and reestablishment of photosynthetic function over time. This ability to regain homeostasis appears to be linked to efficient post-translational regulation and the fine-tuning of defense activation, rather than its mere intensity. The contrasting responses of these two cultivars illustrate how the outcome of FD-infection is determined not only by the magnitude of defense responses but also by the capacity of the host to dynamically remodel its proteome and signaling networks.

#### 4.1.5. Proteome Changes Under *Xylella fastidiosa* Infection

Grapevine Pierce’s disease (PD), caused by the xylem-limited bacterium *Xylella fastidiosa*, represents a systemic vascular disorder of grapevine that severely disrupts water and nutrient transport. The pathogen is transmitted by xylem-feeding insects, primarily sharpshooters and spittlebugs, and represents a major quarantine threat in many viticultural regions. Unlike foliar pathogens, *X. fastidiosa* colonizes the xylem vessels, triggering occlusions, embolisms, and hydraulic dysfunction that ultimately lead to leaf scorch and vine decline. Thus, disease symptoms largely result from vascular failure and water stress rather than direct destruction of leaf tissues. Proteomic investigations have provided crucial insights into the biochemical processes underlying this vascular pathology and have revealed clear differences between susceptible and tolerant *Vitis* species [78,79,80,81,82]. Because the pathogen remains confined to the xylem, many systemic responses observed in leaves reflect secondary effects of hydraulic dysfunction and altered nutrient transport.

In *Vitis vinifera* cultivars susceptible to PD, proteomic analyses of xylem sap and stem tissues revealed a profound reorganization of the vascular proteome following bacterial colonization. Quantitative LC–MS/MS and 2-DE profiling identified the depletion of proteins involved in cell wall remodeling and water transport, such as expansins, xyloglucan endotransglycosylase/hydrolases, and aquaporins, indicating a loss of xylem integrity and vessel obstruction by bacterial biofilm and tyloses [80]. At the same time, strong induction of pathogenesis-related (PR) proteins was observed, including PR-1, PR-2 β-1,3-glucanases, PR-3 and PR-4 chitinases, and PR-5 thaumatin-like proteins, highlighting the activation of innate immune responses within the xylem [78,80,81]. Other defense-related proteins, such as lipid transfer proteins, germin-like oxalate oxidases, and class III peroxidases, also accumulated, contributing to cell wall strengthening and oxidative burst responses.

Proteins associated with oxidative stress management, including superoxide dismutase, glutathione S-transferase, and peroxiredoxins, were upregulated, consistent with an intense redox imbalance in infected vascular tissues [80,81]. However, in fully susceptible *V. vinifera*, these responses appeared insufficient to contain bacterial proliferation, and chronic oxidative damage likely contributed to tissue necrosis. Metabolic enzymes, such as enolase, glyceraldehyde-3-phosphate dehydrogenase, and malate dehydrogenase, were generally downregulated, indicating suppression of primary metabolism and energy production typical of vascular stress and impaired hydraulic conductivity [80].

Comparative proteomics between PD-tolerant *Vitis* species (*V. arizonica*, *V. aestivalis*, and *V. rotundifolia*) and susceptible *V. vinifera* revealed distinct proteome signatures associated with tolerance [78,79,81]. These differences highlight the importance of preformed defense capacity and vascular resilience in determining disease outcome. Tolerant genotypes displayed constitutively higher basal levels of several PR proteins, particularly chitinases, β-1,3-glucanases, and thaumatin-like proteins, as well as enzymes involved in lignin biosynthesis, including peroxidases and phenylalanine ammonia-lyase [78,79]. This preformed defense potential likely limits bacterial spread by reinforcing vessel walls and restricting pathogen movement. Upon infection, tolerant vines exhibited rapid induction of reactive oxygen species–detoxifying enzymes such as ascorbate peroxidase, catalase, and glutathione peroxidase, along with stress signaling components including HSP70 and HSP90 [79]. Interestingly, these genotypes maintained stable levels of metabolic enzymes such as enolase and triosephosphate isomerase, suggesting that tolerance involves preserving metabolic homeostasis while activating defense. Furthermore, elevated levels of polygalacturonase-inhibiting proteins and protease inhibitors supported the concept of localized defense preventing systemic xylem degradation. Tolerance in these species is generally associated with restriction of bacterial spread rather than complete pathogen elimination [79,81].

Structural and computational analyses of the xylem proteome emphasized the dominance of PR proteins, glycosyl hydrolases, chitinases, β-1,3-glucanases, and lectin-like proteins, all possessing domains involved in carbohydrate binding, protease inhibition, and signal perception [81]. These proteins are secretory, highly glycosylated, and structurally stable under alkaline and oxidative xylem conditions, implying evolutionary adaptation for persistent vascular defense. Recent advances integrating deep learning neural network prediction have expanded xylem proteome coverage, identifying previously undetected low-abundance proteins, including PR peptides, metallothioneins, and redox-active enzymes that likely contribute to early pathogen recognition and detoxification processes [82].

**Figure 7:**
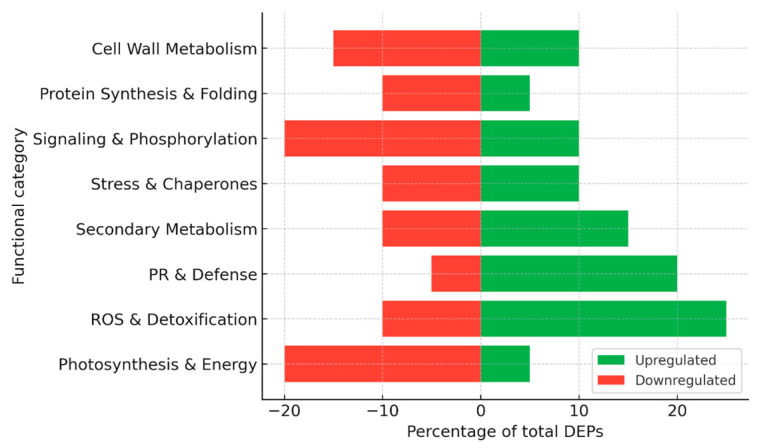
Distribution of differentially expressed proteins (DEPs) across functional categories in grapevine stems and xylem during infection by *Xylella fastidiosa*. Proteins were classified according to their biological functions. Values represent the proportion of upregulated and downregulated proteins within each category, normalized to 100% for the dataset. These values reflect the distribution of functional categories rather than absolute protein abundance. DEPs were extracted from literature sources, and no cross-study statistical analysis was performed due to dataset heterogeneity.

Proteomic approaches are particularly valuable for studying PD because symptom development results from complex host responses to vascular colonization rather than localized infection. Together, these studies demonstrate that *X. fastidiosa* infection induces a systemic reprogramming of the grapevine vascular proteome (Figure 7). In susceptible *V. vinifera*, infection results in a severe downregulation of metabolic and structural proteins and a delayed, ineffective activation of defense mechanisms, culminating in uncontrolled oxidative stress and xylem collapse. In tolerant *Vitis* species, by contrast, constitutive and inducible defense proteins reinforce vessel walls, maintain redox homeostasis, and preserve essential metabolic activity, thereby restricting pathogen colonization. The xylem proteome therefore acts not merely as a passive conduit for water and solutes but as a dynamic biochemical defense network whose stability and flexibility determine the outcome of *X. fastidiosa* infection. Understanding these mechanisms is essential for breeding and biotechnological strategies aimed at improving resistance to *X. fastidiosa*.

#### 4.1.6. Integrative Analysis of Proteomic Adaptations to Biotic Stress in *Vitis* Species

The integration of proteomic data from multiple grapevine–pathogen systems reveals a high degree of plasticity of the *Vitis* proteome, enabling both rapid localized responses and long-term systemic adjustments (Table 1). These pathogens represent diverse lifestyles, including biotrophic foliar pathogens, vascular bacteria, phytoplasmas, and wood-decaying fungi, each imposing distinct physiological constraints on the host. Despite these differences—ranging from foliar oomycetes (*Plasmopara viticola*), fungal biotrophs (*Erysiphe necator*), and vascular bacteria (*Xylella fastidiosa*) to phytoplasmas and fungal consortia associated with Esca disease—proteomic responses consistently converge on a limited set of functional processes, particularly those related to energy metabolism, redox regulation, and defense signaling [62,63,64,65,66,67,68,69,70,71,72,73,74,75,76,77,78,79,80,81,82].

Acute foliar infections, such as those caused by *P. viticola* and *E. necator*, primarily target photosynthetically active tissues and induce an early and transient suppression of proteins involved in photosynthesis and primary metabolism, including RuBisCO, chlorophyll a/b-binding proteins, and ATP synthase subunits. This is accompanied by rapid induction of pathogenesis-related proteins (PR-2 β-1,3-glucanases, PR-3 chitinases, PR-5 thaumatin-like proteins), oxidative stress enzymes (superoxide dismutase, peroxidases, ascorbate peroxidase, glutathione S-transferase), and molecular chaperones (HSP70, HSP90), reflecting activation of defense signaling pathways [62,63,64,65,66,67,68,69,70,71,72,73]. Together, these changes indicate a rapid metabolic reprogramming in which energy-consuming processes are downregulated while defense and redox systems are prioritized. In resistant genotypes or elicitor-treated plants, this reprogramming is accompanied by maintenance of metabolic balance and activation of secondary metabolism, particularly phenylpropanoid and jasmonate pathways, ensuring both energy supply and production of defensive compounds such as lignin and stilbenes [65,66,67,68,69,70].

In contrast, chronic vascular diseases, including Flavescence dorée and *X. fastidiosa* infection, result in long-lasting systemic proteome reorganization. In susceptible cultivars such as Nebbiolo, these interactions are characterized by collapse of photosynthetic capacity and persistent redox imbalance, whereas tolerant cultivars (e.g., Barbera or resistant *Vitis* species) exhibit partial restoration of photosynthetic machinery and stabilization of ROS homeostasis. This indicates that tolerance to vascular pathogens depends not only on activation of defense responses, but also on the ability to re-establish metabolic equilibrium during prolonged stress. Phosphoproteomic and xylem proteome studies further suggest that post-translational regulation plays a key role in coordinating metabolic recovery and maintaining vascular integrity. Constitutive and induced accumulation of PR proteins, chitinases, β-1,3-glucanases, peroxidases, and lipid transfer proteins contributes to reinforcement of xylem cell walls, while differential phosphorylation of metabolic and signaling proteins supports long-term adaptation [76,77,78,79,80,81,82].

The Esca complex further illustrates the complexity of grapevine responses to multi-pathogen systems. In this case, necrotrophic fungi induce localized cell wall degradation and toxin-associated oxidative stress, which is counteracted by increased abundance of detoxifying enzymes, lignin biosynthetic enzymes, and stress-related chaperones [74,75]. These responses highlight the importance of coordinated structural reinforcement and detoxification processes, rather than reliance on a single dominant defense pathway.

Overall, comparative proteomic evidence indicates that resistance and tolerance in grapevine are governed by two complementary and interconnected principles: (i) rapid activation of defense-related proteins, including PR proteins and redox enzymes, and (ii) the capacity to maintain or restore metabolic equilibrium during infection. The balance between these processes emerges as a key determinant of disease outcome across diverse pathogen systems. This dual strategy underpins the ability of *Vitis* species to cope with biotic stress and provides a mechanistic framework for the development of breeding and biotechnological approaches aimed at improving durable disease resistance.

### 4.2. Leaf Proteomics Under Abiotic Stress

#### 4.2.1. Water Stress

Water deficit is one of the major abiotic factors limiting grapevine productivity and quality. Proteomic studies show that drought reprograms leaf metabolism across photosynthesis, carbon and energy pathways, protein fate, and redox homeostasis.

Early time-course work in Cabernet Sauvignon revealed that a progressive water deficit triggers extensive proteome shifts even before visible declines in shoot growth or photosynthesis occur. Early-responding proteins were mainly associated with photosynthesis, glycolysis, translation, antioxidant defense, and growth-related processes, while later stages showed changes in transport, photorespiration, and amino-acid and carbohydrate metabolism, illustrating a tightly regulated, multistage stress response [83].

A comparative study of two wild *Vitis sylvestris* accessions (Tebaba and Houamdia) identified 66 drought-responsive proteins, of which 48 were genotype-specific. In the tolerant accession Tebaba, proteins related to antioxidant defense (ascorbate peroxidase, abscisic stress ripening protein 2, glutaredoxin GRXS17), protein quality control (26S protease, ClpA), and signal transduction (14–3–3-like protein) were upregulated. Additionally, RuBisCO activase, ribulose-1,5-bisphosphate carboxylase, and RNA-binding proteins were elevated, maintaining photosynthetic and translational activity under stress. In contrast, these proteins were downregulated in the drought-sensitive Houamdia, emphasizing genotype-dependent resilience [84].

Under long-term drought conditions, *V. vinifera* leaves displayed marked reorganization of the proteome. Using 2-DE/MALDI-TOF/TOF, the most numerous changes occurred in proteins linked to energy and carbohydrate metabolism, stress and defense, and genetic information processing. Many glycolytic enzymes decreased, while proteins involved in photosynthesis and photorespiration increased, revealing a complex metabolic adjustment that helps balance energy production and ROS control during extended drought [85]. Previous cultivar comparisons further demonstrated that responses differ between grapevine genotypes. In studies of Chardonnay and Cabernet Sauvignon under water deficit (and salinity, with only the drought component considered here), both cultivars exhibited broad proteomic adjustments encompassing photosynthesis, energy metabolism, protein fate, and detoxification pathways, confirming the multifaceted adaptive nature of *Vitis vinifera* under water limitation [86].

For contrast within abiotic water stress, waterlogging of the SO4 rootstock (*V. berlandieri* × *V. riparia*) elicited a distinct proteomic response compared to drought. TMT-based LC–MS/MS profiling identified over 5500 proteins, with hundreds of differentially expressed proteins (DEPs) enriched in glutathione metabolism, carbon fixation, amino-acid biosynthesis, and protein processing in the endoplasmic reticulum. Upregulation of glutathione S-transferase, pyruvate kinase, and alcohol dehydrogenase reflected adaptation to hypoxia, while downregulation of ribosomal proteins suggested transient inhibition of protein synthesis [87].

**Figure 8:**
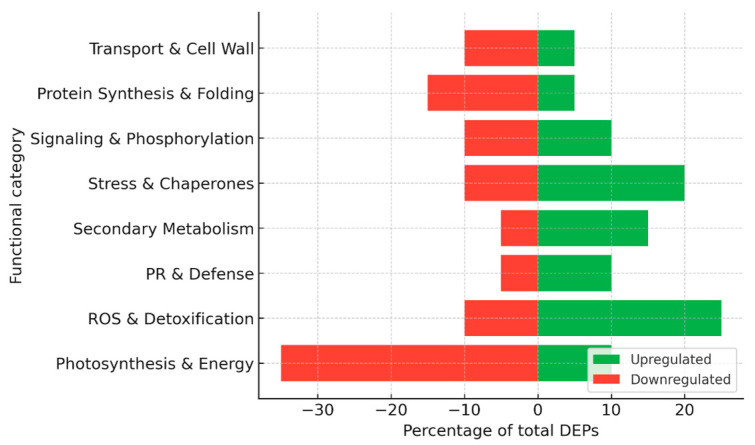
Distribution of differentially expressed proteins (DEPs) across functional categories in grapevine leaves under water deficit conditions. Proteins were classified according to their biological functions. Values represent the proportion of upregulated and downregulated proteins within each category, normalized to 100% for the dataset. These values reflect the distribution of functional categories rather than absolute protein abundance. DEPs were extracted from literature sources, and no cross-study statistical analysis was performed due to dataset heterogeneity.

Together, these studies demonstrate that grapevine leaf proteomes undergo coordinated adjustments to maintain cellular redox balance, energy metabolism, and protein homeostasis under water stress (Figure 8). Such proteomic insights underline the central roles of antioxidant enzymes, molecular chaperones, and carbohydrate-metabolic enzymes in drought tolerance and provide valuable markers for breeding programs targeting improved stress resilience.

#### 4.2.2. Salinity Stress

Salinity imposes both osmotic and ionic stress on grapevine, leading to alterations in energy metabolism, protein stability, and antioxidant defense. Proteomic investigations have revealed that the response to salt stress is strongly genotype-dependent, involving extensive reorganization of photosynthetic, stress-related, and detoxification proteins.

In the comparative study by Vincent et al., *Vitis vinifera* cultivars with contrasting salt tolerance displayed clear differences in their proteomic adjustment to NaCl exposure. In the salt-tolerant cultivar, key photosynthetic enzymes such as RuBisCO large subunit, glyceraldehyde-3-phosphate dehydrogenase, and phosphoglycerate kinase were maintained or even upregulated, indicating preservation of primary carbon metabolism under moderate stress. In contrast, the sensitive cultivar exhibited strong downregulation of photosynthetic proteins together with upregulation of heat shock proteins (HSP70, HSP90), dehydrins, and chaperonins, reflecting proteome protection at the expense of carbon assimilation. The tolerant cultivar also induced peroxiredoxin, superoxide dismutase (SOD), and ascorbate peroxidase (APX), highlighting a more efficient ROS detoxification system compared with the sensitive genotype [86].

Proteomic profiling of the Tunisian cultivar Razegui under salt stress confirmed substantial changes in both metabolic and structural proteins. Using two-dimensional electrophoresis and MALDI-TOF/TOF, Jellouli et al. identified major variations in photosynthesis-related proteins, ATP synthase β-subunit, oxygen-evolving enhancer protein, and chlorophyll a/b binding protein, all of which were downregulated in response to NaCl. Conversely, proteins associated with stress protection, including late embryogenesis abundant (LEA) proteins, dehydrins, and heat shock proteins, were strongly upregulated, suggesting a compensatory mechanism to preserve protein folding and membrane stability. Additionally, enzymes from the glycolytic pathway (enolase, fructose-bisphosphate aldolase) and TCA cycle (malate dehydrogenase) increased in abundance, reflecting a metabolic shift toward respiration to meet energy demands under ionic stress [88].

In a complementary study, Jellouli et al. examined the proteomic and transcriptomic regulation of PR-10 proteins in grapevine under salinity. PR-10 accumulated in salt-stressed leaves and was accompanied by increased expression of several detoxification and signaling proteins, including glutathione S-transferase (GST), protein disulfide isomerase (PDI), and 14–3–3-like proteins. These findings linked PR-10 induction with broader stress signaling and antioxidant activity, suggesting that PR-10 may act as a molecular hub integrating redox balance and defense regulation [89].

Further proteomic insights were provided by Patil et al., who compared salt responses in own-rooted Thompson Seedless and 110R-grafted plants using a label-free LC–MS/MS approach. More than 800 differentially expressed proteins were identified. In own-rooted plants, NaCl treatment led to strong downregulation of photosynthetic and Calvin-cycle proteins, including RuBisCO activase and ferredoxin–NADP^+^ reductase, whereas 110R-grafted plants showed enhanced abundance of antioxidant enzymes, lignin biosynthesis enzymes, and secondary metabolism proteins. The grafted combination also displayed increased levels of aquaporins and ABC transporters, which may facilitate ion compartmentalization and osmotic adjustment under salt exposure [90].

**Figure 9:**
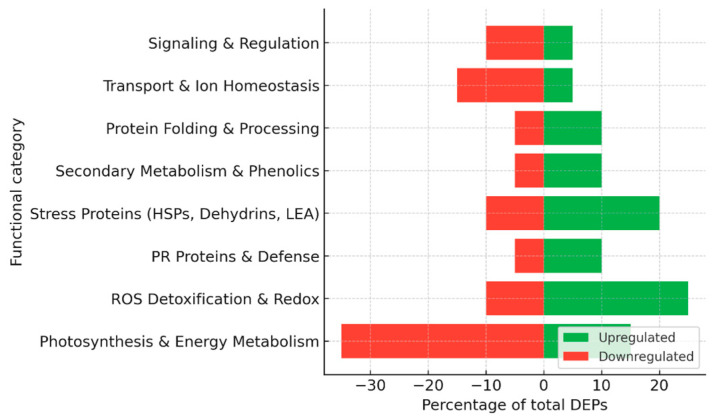
Distribution of differentially expressed proteins (DEPs) across functional categories in grapevine leaves under salinity stress. Proteins were classified according to their biological functions. Values represent the proportion of upregulated and downregulated proteins within each category, normalized to 100% for the dataset. These values reflect the distribution of functional categories rather than absolute protein abundance. DEPs were extracted from literature sources, and no cross-study statistical analysis was performed due to dataset heterogeneity.

Altogether, proteomic analyses highlight a core network of redox-regulatory proteins (SOD, APX, peroxiredoxin), molecular chaperones (HSPs, dehydrins), and metabolic enzymes (glycolytic and TCA cycle components) as central elements of grapevine salt stress tolerance. Differential regulation of photosynthetic machinery, combined with induction of PR proteins and detoxification pathways, appears to determine the contrasting capacity of grapevine cultivars and grafted combinations to maintain metabolic homeostasis under saline conditions (Figure 9).

#### 4.2.3. Heat Stress

High temperature represents one of the most critical abiotic stress factors limiting grapevine productivity and quality. Recent proteomic and multi-omics analyses have revealed that exposure to heat induces profound remodeling of grapevine leaf metabolism, protein modification, and signaling networks, with a strong emphasis on redox balance, protein stability, and transcriptional regulation.

In an iTRAQ-based proteomic analysis of Cabernet Sauvignon, Liu et al. identified more than 2000 proteins, of which 161 were significantly altered during heat stress and subsequent recovery. The early response phase was characterized by strong accumulation of heat shock proteins (HSP70, HSP90, small HSPs), chaperonins, and proteasome-related subunits, indicating activation of the protein quality-control system. Proteins associated with ROS detoxification, including superoxide dismutase (SOD), ascorbate peroxidase (APX), thioredoxin peroxidase, and glutathione S-transferase (GST), were also upregulated, suggesting reinforcement of antioxidant defenses. In contrast, components of the photosynthetic apparatus—notably RuBisCO large subunit, RuBisCO activase, oxygen-evolving enhancer (OEE), and chlorophyll a/b-binding proteins—were downregulated, consistent with photosynthetic inhibition under high temperature. During recovery, several metabolic enzymes such as fructose-bisphosphate aldolase, enolase, and malate dehydrogenase regained abundance, indicating restoration of carbon and energy metabolism once the stress subsided [91].

The integration of transcriptomics and proteomics by Jiang et al. expanded this picture by showing that high temperature triggers both transcriptional and post-transcriptional reprogramming. Heat exposure induced alternative splicing in genes encoding HSPs, protein phosphatases 2C (PP2Cs), calmodulin-binding proteins, and transcription factors (HSFs, WRKYs, bZIPs). At the proteomic level, the authors observed increased abundance of HSP101, HSP90, mitochondrial chaperone GrpE, and HSP-interacting protein Sti1, confirming activation of the cytosolic and organellar chaperone network. Simultaneously, accumulation of enzymes from the ascorbate–glutathione (AsA-GSH) cycle reinforced oxidative-stress tolerance. Heat also promoted expression of lipid metabolism enzymes, including glycerol-3-phosphate acyltransferase and lipoxygenase, suggesting remodeling of membrane composition to maintain fluidity under elevated temperatures [92].

At the post-translational level, Liu et al. provided new insights by combining phosphoproteomics and acetylproteomics of grape leaves exposed to high temperature. They identified more than 6000 phosphorylation and 3500 acetylation sites, revealing extensive modification of proteins involved in photosynthesis, signal transduction, energy metabolism, and protein turnover. Notably, RuBisCO activase, ATP synthase β-subunit, and several HSPs were both phosphorylated and acetylated, implying that dynamic regulation of these proteins fine-tunes photosynthetic recovery and stress adaptation. The study also highlighted hyper-acetylation of histones H3/H4, elongation factors, and RNA-binding proteins, linking epigenetic and translational control to heat tolerance. Enhanced phosphorylation of calcium-dependent protein kinases (CDPKs) and mitogen-activated protein kinases (MAPKs) pointed to rapid signal amplification following temperature rise [93].

**Figure 10:**
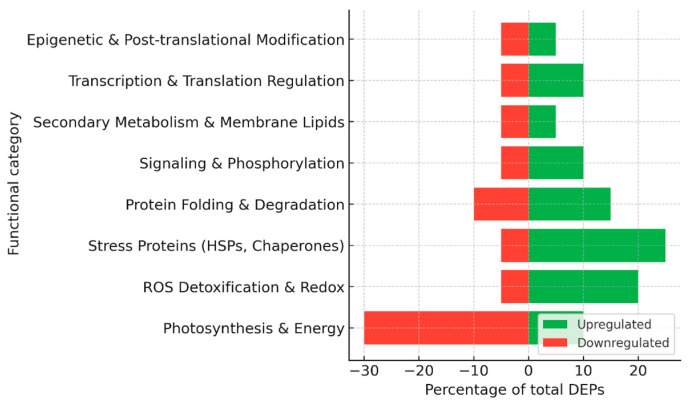
Distribution of differentially expressed proteins (DEPs) across functional categories in grapevine leaves under heat stress. Proteins were classified according to their biological functions. Values represent the proportion of upregulated and downregulated proteins within each category, normalized to 100% for the dataset. These values reflect the distribution of functional categories rather than absolute protein abundance. DEPs were extracted from literature sources, and no cross-study statistical analysis was performed due to dataset heterogeneity.

Collectively, proteomic and post-translational data demonstrate that grapevine leaves respond to heat stress through a coordinated program integrating chaperone-mediated protein protection, ROS homeostasis, membrane remodeling, and signal transduction (Figure 10). The reversible suppression of photosynthetic proteins and the activation of repair systems during recovery illustrate the remarkable plasticity of the grapevine proteome in maintaining metabolic and redox equilibrium under fluctuating temperature regimes.

#### 4.2.4. Other Abiotic Stresses

In addition to drought, salinity, and temperature, grapevine leaves experience diverse environmental and chemical challenges that trigger specific proteomic adjustments. Studies addressing photooxidative stress, herbicide exposure, photoperiod-induced dormancy, and hormonal signaling have revealed distinct yet overlapping molecular strategies that maintain cellular homeostasis and redox balance.

Exposure to high light irradiance during the transition from heterotrophy to autotrophy was shown to cause substantial remodeling of the leaf proteome. Using two-dimensional electrophoresis coupled with MALDI-TOF/TOF, Nilo-Poyanco et al. identified major changes in photosynthetic and redox-related proteins. Upregulated proteins included oxygen-evolving enhancer proteins (OEE1, OEE2), chlorophyll a/b binding proteins, cytochrome b6-f complex subunits, and ferrodoxin–NADP^+^ reductase, indicating reinforcement of the photosynthetic apparatus under high photon flux. Concurrently, proteins involved in ROS detoxification such as superoxide dismutase (SOD), ascorbate peroxidase (APX), glutathione S-transferase (GST), and 2-Cys peroxiredoxin accumulated to counteract photooxidative stress. Enhanced expression of carbonic anhydrase and triosephosphate isomerase suggested an acceleration of carbon assimilation and energy turnover. In contrast, a small subset of metabolic proteins linked to nitrogen assimilation and translation decreased, reflecting resource reallocation toward photoprotection and antioxidant defense [94].

Proteomic analysis of herbicide stress revealed similar emphasis on oxidative stress mitigation and detoxification mechanisms. Castro et al. analyzed *Vitis vinifera* leaves exposed to glyphosate and glufosinate and identified 36 differentially expressed proteins using 2-DE and LC–MS/MS. Enzymes related to redox homeostasis—notably glutathione S-transferase, catalase, and ascorbate peroxidase—were strongly induced, alongside pathogenesis-related (PR) proteins such as β-1,3-glucanase and chitinase, suggesting that herbicide exposure triggers a defense-like response. Moreover, increases in S-adenosylmethionine synthase and methionine adenosyltransferase indicated enhanced methylation and detoxification activity. Downregulated proteins included RuBisCO large subunit, oxygen-evolving enhancer proteins, and ATP synthase subunits, consistent with inhibition of photosynthesis and energy metabolism under oxidative chemical stress [95].

In *Vitis riparia*, photoperiod-induced growth cessation and dormancy transitions were studied by Victor et al. using a comparative proteomic approach to examine shoot and leaf tissues during long-day to short-day transitions. The analysis revealed an early increase in dehydrins, late embryogenesis abundant (LEA) proteins, and heat shock proteins (HSP70, HSP90), reflecting preparation for cold acclimation. Simultaneously, S-adenosylmethionine synthetase, adenosine kinase, and methionine synthase were upregulated, suggesting reinforcement of methyl group metabolism and polyamine biosynthesis, processes essential for stress tolerance and growth arrest. Several enzymes involved in phenylpropanoid biosynthesis, including phenylalanine ammonia-lyase (PAL) and caffeic acid O-methyltransferase (COMT), also increased, consistent with lignin and flavonoid accumulation during the onset of dormancy. Photosynthetic and ribosomal proteins decreased, indicating a shift from growth to stress-protective metabolism [96].

Finally, Rattanakan et al. investigated abscisic acid (ABA)-induced proteomic and phosphoproteomic reprogramming in Cabernet Sauvignon leaves. Within 2 h of root ABA exposure, 1011 proteins and 219 phosphoproteins were identified, of which 20 changed in abundance and 11 showed altered phosphorylation status. Downregulated proteins included light-harvesting complex proteins (Lhca2), oxygen-evolving enhancer protein 3, and several ribosomal proteins, reflecting suppression of photosynthesis and translation. Conversely, ABA increased the abundance of NADP-malic enzyme, 6-phosphogluconate dehydrogenase, oxygen-evolving enhancer protein (Q6XGX7), and voltage-dependent anion channels, all linked to redox balance and energy metabolism. Phosphorylation motifs characteristic of SnRK2 kinase substrates were detected, indicating canonical ABA signaling cascades controlling stress adaptation [97].

#### 4.2.5. Summary of Abiotic Stress–Induced Proteomic Responses

Proteomic studies have revealed that grapevine leaves possess exceptional metabolic flexibility, enabling adaptation to a wide range of abiotic stresses, including water deficit, salinity, high temperature, light excess, herbicide exposure, photoperiod-induced dormancy, and hormonal regulation (Table 2). Despite differences in the initial stimuli, these stressors induce overlapping proteomic patterns that converge on a limited number of adaptive processes aimed at preserving cellular integrity, redox homeostasis, and energy balance.

A key unifying feature across abiotic stresses is the reorganization of photosynthetic metabolism. Under drought and salinity, major photosynthetic proteins such as the RuBisCO large and small subunits, chlorophyll a/b-binding proteins, oxygen-evolving enhancer proteins, and ATP synthase subunits are consistently downregulated, reflecting suppression of carbon fixation and photochemical activity to minimize photooxidative stress [85,86,88,90]. However, during recovery following drought or heat exposure, components of the photosynthetic apparatus—including RuBisCO activase and ferredoxin–NADP^+^ reductase—show partial restoration, indicating that photosynthetic regulation is dynamic and reversible, and closely linked to stress duration and intensity [86,91]. In parallel, all stresses trigger the accumulation of antioxidant and redox-regulatory enzymes, highlighting oxidative stress as a common secondary constraint. Enzymes such as superoxide dismutase (SOD), ascorbate peroxidase (APX), glutathione S-transferase (GST), thioredoxin peroxidase, and peroxiredoxins are strongly upregulated in salinity-, heat-, and light-stressed leaves, forming an integrated detoxification network that limits reactive oxygen species accumulation [88,91,93]. Additional enzymes, including NADP-malic enzyme, 6-phosphogluconate dehydrogenase, and catalase, contribute to NADPH regeneration and maintenance of cellular redox balance, thereby sustaining metabolic stability during prolonged stress [85,93]. Together, these changes reflect a coordinated adjustment in which photosynthetic activity is reduced while redox buffering capacity is enhanced to prevent cellular damage.

Another central mechanism involves maintenance of protein homeostasis through molecular chaperones and proteolytic systems. The accumulation of HSP70, HSP90, small HSPs, and chaperonins is consistently observed under drought, salinity, and heat stress, reflecting increased protein folding capacity and prevention of aggregation under denaturing conditions [90,92,93]. Concomitant induction of protein disulfide isomerase and components of the ubiquitin–proteasome system in heat- and herbicide-treated tissues further highlights the importance of regulated protein turnover in maintaining proteome integrity [93,95]. These responses indicate that stabilization and controlled degradation of proteins are essential for preserving functional proteomes under stress.

Metabolic reprogramming toward secondary metabolism and osmoprotection represents another conserved response. Under salinity and photoperiod-induced dormancy, increased abundance of phenylalanine ammonia-lyase (PAL), caffeic acid O-methyltransferase (COMT), and stilbene synthase (STS) indicates enhanced synthesis of phenolic compounds involved in antioxidant defense and structural reinforcement [83,85,90]. The accumulation of S-adenosylmethionine synthase and methionine adenosyltransferase further suggests that methyl group metabolism and polyamine biosynthesis contribute to osmotic adjustment and redox homeostasis [90]. These metabolic shifts support both chemical defense and stabilization of cellular structures under stress conditions.

At the regulatory level, proteomic and phosphoproteomic data demonstrate that signal transduction and transcriptional control are fine-tuned through post-translational modifications. In heat-stressed leaves, activation of mitogen-activated protein kinases (MAPKs), calcium-dependent protein kinases (CDPKs), and heat shock transcription factors (HSFs) promotes transcription of stress-responsive genes [91]. Similarly, in ABA-treated leaves, phosphorylation motifs associated with SnRK2 kinase activity, together with changes in ABC transporters and protein phosphatases 2C (PP2Cs), confirm the activation of canonical ABA-dependent signaling pathways [97]. In addition, extensive acetylation and phosphorylation of photosynthetic, ribosomal, and nuclear proteins indicate that chromatin organization and translational efficiency are directly modulated in response to stress [91,97], linking post-translational regulation to functional adaptation of the proteome.

Overall, these proteomic data indicate that grapevine leaves respond to abiotic stress through a conserved and coordinated network of adaptive processes. This network can be summarized into five principal strategies: (1) transient downregulation and subsequent recovery of photosynthesis; (2) activation of antioxidant and detoxification pathways to mitigate oxidative damage; (3) stabilization and controlled turnover of proteins via molecular chaperones and proteolytic systems; (4) metabolic reprogramming toward phenolic and osmoprotective compounds; and (5) multilayered regulation through post-translational modifications and signaling pathways. The effectiveness of the stress response depends on the coordination and timing of these processes, which together maintain metabolic homeostasis under fluctuating environmental conditions. Such integrative proteomic understanding provides a foundation for identifying candidate proteins and pathways that can be targeted to enhance abiotic stress tolerance in grapevine breeding and biotechnological applications.

#### 4.2.6. Integrative View of Grapevine Leaf Proteome Responses

Despite the diversity of biotic and abiotic stressors affecting grapevine leaves, proteomic studies reveal a highly conserved set of adaptive responses that converge on a limited number of core biological processes. Both pathogen-induced and abiotic stresses consistently trigger a reorganization of photosynthetic metabolism, characterized by downregulation of carbon fixation and energy production, coupled with activation of redox-regulatory and defense-related systems.

Central to these responses is the modulation of reactive oxygen species (ROS) homeostasis, where enzymes such as superoxide dismutase, ascorbate peroxidase, glutathione S-transferase, and peroxiredoxins form a coordinated detoxification network that operates across stress types. In parallel, molecular chaperones (HSP70, HSP90, small HSPs) and proteolytic systems maintain protein stability and turnover, ensuring functional integrity of the proteome under both transient and prolonged stress conditions.

A key distinguishing factor between effective and ineffective responses lies in the ability to maintain metabolic balance. In both biotic and abiotic contexts, susceptible or stressed tissues often exhibit strong suppression of photosynthesis without sufficient compensatory mechanisms, leading to energy limitation. In contrast, resistant genotypes or stress-tolerant conditions are associated with partial preservation or rapid restoration of primary metabolism, combined with activation of secondary metabolic pathways such as phenylpropanoid and jasmonate signaling.

Post-translational regulation further contributes to this adaptive capacity by fine-tuning signaling networks and metabolic pathways, allowing rapid adjustment to changing environmental conditions.

Collectively, these findings indicate that grapevine leaf responses are governed by a coordinated balance between defense activation, redox control, and maintenance of metabolic homeostasis. This balance represents a unifying principle underlying proteome reprogramming across diverse stress conditions and provides a conceptual framework for understanding leaf resilience in grapevine.

## 5. Bud Proteomics

Proteomic analysis of grapevine buds remains relatively limited compared to other plant organs, with only a small number of studies addressing the molecular basis of bud dormancy, development, and reactivation, particularly in comparison with leaf and berry proteomics. Despite this limited dataset, available studies provide valuable insights into the molecular mechanisms underlying bud physiology and reveal a highly coordinated proteomic response to environmental cues. The grapevine bud is a highly specialized meristematic structure whose dormancy, cold acclimation, and subsequent reactivation determine the seasonal growth cycle. Proteomic studies indicate that these processes are governed by coordinated changes in metabolic activity, redox homeostasis, cytoskeletal organization, and signaling pathways, rather than by simple growth arrest.

Dormancy induction is closely associated with photoperiod-dependent metabolic reprogramming. Early physiological and protein profiling studies demonstrated that exposure to short photoperiods leads to reduced respiration, decreased bud water content, and accumulation of specific low-molecular-weight polypeptides of approximately 17 and 19 kDa during dormancy establishment [98]. Subsequent proteomic analyses revealed decreased abundance of proteins associated with photosynthesis and primary carbon metabolism, including enzymes of the Calvin cycle and carbohydrate metabolism, reflecting suppression of biosynthetic activity during endodormancy [99].

At the same time, dormancy is accompanied by activation of redox- and stress-related proteins. Increased abundance of superoxide dismutase (SOD), class III peroxidases, and various dehydrogenases has been reported, indicating that oxidative stress signaling, and ROS homeostasis are central components of endodormancy commitment [99]. These proteins contribute not only to detoxification but also to signaling processes regulating dormancy progression.

Structural reorganization of the bud is another critical feature revealed by proteomic studies. Analysis of cytoskeletal proteins demonstrated differential accumulation of α- and β-tubulin isoforms during bud development, with more acidic isoforms predominating in dormant buds and distinct isoform patterns associated with actively growing tissues [100]. These changes reflect cytoskeletal remodeling required to maintain meristem integrity during dormancy and to enable rapid reactivation during bud burst.

Dormancy release is characterized by a large-scale reactivation of metabolic and biosynthetic processes. Proteomic studies using hydrogen cyanamide (HC) have identified extensive changes in protein abundance, including upregulation of enzymes involved in carbohydrate metabolism such as sucrose synthase and hexokinase, as well as proteins associated with protein synthesis, including elongation factors EF-1α and EF-2 [101,102]. In parallel, proteins involved in redox regulation and stress response, including glutathione S-transferases (GSTs), peroxiredoxins, and peroxidases, are strongly induced, indicating coordinated activation of antioxidant systems and ROS-mediated signaling. Enrichment of ribosomal proteins and translation-related factors further supports activation of the protein synthesis machinery during bud burst. Proteomic and integrative analyses have also identified MAPK cascade components and SnRK kinases as key regulators linking ROS signaling, hormonal control, and cell cycle re-entry.

In contrast to dormancy-focused studies, proteomic investigations of cold acclimation in grapevine buds remain limited but provide important insights into freezing tolerance mechanisms. A comparative iTRAQ-based analysis of *Vitis amurensis* and *V. vinifera* buds identified 472 and 713 differentially abundant proteins, respectively, during seasonal cold acclimation, with 235 proteins shared between the two species [103]. These shared proteins were primarily associated with protein folding, stress response, and carbohydrate metabolism, indicating a conserved core acclimation response. Notably, species-specific differences were observed in the regulation of phenylpropanoid metabolism. In the cold-tolerant *V. amurensis*, key enzymes such as phenylalanine ammonia-lyase (PAL), cinnamoyl-CoA reductase 1 (CCR1), and shikimate O-hydroxycinnamoyl transferase (HCT) accumulated during cold acclimation, whereas their abundance decreased in *V. vinifera*. These enzymes are central to lignin biosynthesis and secondary cell wall formation, suggesting enhanced structural reinforcement as a key mechanism of freezing tolerance. Additional proteins identified during cold acclimation include molecular chaperones involved in protein folding and stabilization, particularly heat shock proteins (HSPs) and related chaperone systems, as well as enzymes associated with carbohydrate metabolism that contribute to osmotic adjustment and maintenance of cellular integrity under low-temperature conditions. The contrasting accumulation patterns between *V. amurensis* and *V. vinifera* highlight divergent adaptive strategies, in which enhanced secondary metabolism and structural stabilization support increased freezing tolerance [103].

Taken together, these studies demonstrate that grapevine bud physiology is governed by a highly coordinated proteomic program involving specific protein groups and pathways (Table 3). Dormancy induction is associated with suppression of photosynthetic metabolism and activation of redox enzymes such as SOD and peroxidases; cold acclimation involves enzymes such as PAL, CCR1, and HCT alongside chaperones and metabolic regulators; and dormancy release is driven by activation of carbohydrate metabolism (sucrose synthase, hexokinase), protein synthesis (elongation factors), and redox regulation (GSTs, peroxiredoxins). This integrated proteomic framework highlights the dynamic nature of bud dormancy and its tight coupling with environmental cues, particularly photoperiod and temperature, which together regulate the balance between survival and growth in grapevine.

## 6. Root Proteomics

Proteomic studies of grapevine roots are still scarce in comparison with those of leaves, berries, and wine, and the currently available knowledge is based on only a few experimental systems. In practice, the field has been developed predominantly by the group of Prinsi and colleagues [104,105,106], who systematically investigated root responses to drought, salinity, and nitrate availability in a limited number of rootstocks, particularly M4 and 101.14, whereas Chen et al. [107] later extended root proteomics to freezing stress in a cold-resistant interspecific hybrid and Cabernet Sauvignon. Accordingly, current data do not yet describe a general grapevine root proteome, but rather a set of genotype- and stress-specific case studies that nonetheless provide valuable mechanistic insight [104,105,106,107].

Despite this limited coverage, the available studies consistently show that root proteome remodeling in *Vitis* involves tightly coordinated changes in carbon and energy metabolism, ion compartmentation, oxidative stress control, protein turnover, and structural adaptation. A recurring theme in tolerant genotypes is the capacity to preserve mitochondrial energy metabolism and redox balance, whereas susceptible genotypes more often show proteotoxic stress, fermentation-like responses, and stronger evidence of tissue damage [104,106].

Under water deficit, the best-characterized system is the comparison between the drought-tolerant rootstock M4 and the more sensitive 101.14. Proteomic and metabolomic analysis showed that drought altered a substantial fraction of the detected proteins and revealed clear differences in root metabolic strategy between the two genotypes. In the more sensitive 101.14, proteins linked to primary metabolism and energy production were more strongly affected, including phosphoglycerate kinase, glyceraldehyde-3-phosphate dehydrogenase, and malate dehydrogenase, consistent with impaired glycolytic and respiratory performance. By contrast, M4 showed stronger maintenance of stress-mitigating proteins, including heat shock proteins HSP70 and HSP90, antioxidant enzymes such as superoxide dismutase, ascorbate peroxidase, and glutathione S-transferases, and stress-associated proteins such as LEA-like proteins. The same study also linked drought tolerance in M4 to osmotic adjustment and better preservation of mitochondrial functionality and root integrity, indicating that drought tolerance in grapevine roots is closely associated with the ability to maintain both metabolic flux and protective protein networks under severe water deficit [106].

The salinity study provides the most detailed protein-level picture currently available for grapevine root responses to ionic stress. Salt exposure increased root Na^+^ and Cl^−^ in both M4 and 101.14, but the two genotypes differed in how they handled ion sequestration and the energetic cost of stress adaptation. Western blot analyses showed no clear changes in NHX1 or vacuolar H^+^-PPase abundance, whereas the abundance of one V-ATPase subunit E isoform decreased under salt stress in 101.14 but remained stable and constitutively higher in M4, supporting the interpretation that M4 better sustains the proton gradient required for vacuolar Na^+^ compartmentation [104].

At the level of carbon and energy metabolism, salt stress caused strong but contrasting proteomic shifts. In 101.14, sucrose synthase showed a dramatic increase, pyruvate kinase and the E1 β-subunit of mitochondrial pyruvate dehydrogenase increased, but fructose-bisphosphate aldolase and glyceraldehyde-3-phosphate dehydrogenase decreased, together with the dihydrolipoamide acetyltransferase component of the pyruvate dehydrogenase complex. Alcohol dehydrogenase and pyruvate decarboxylase 1 also accumulated specifically in 101.14, suggesting activation of fermentative metabolism as a response to low ATP availability. In contrast, M4 showed a more flexible reorganization of energy pathways: although glyceraldehyde-3-phosphate dehydrogenase, fructose-bisphosphate aldolase, and fructokinase decreased, cytoplasmic phosphoglucomutase, pyruvate kinase, three mitochondrial succinate dehydrogenase subunits, and subunit O of mitochondrial ATP synthase increased, indicating better preservation of ATP-generating capacity. NADP-dependent malic enzyme increased in both genotypes, consistent with a demand for NADPH under stress [104].

Salt stress also exposed clear genotype differences in protein turnover, secondary metabolism, and defense. In 101.14, a 26S proteasome non-ATPase regulatory subunit 2 homolog increased, while two chaperonin 60 α2 proteins decreased, suggesting proteotoxic imbalance. M4, in contrast, showed increased elongation factors and 40S ribosomal protein SA, consistent with better maintenance of protein synthesis. In secondary metabolism, cinnamyl alcohol dehydrogenase 8 and chalcone synthase decreased in 101.14, whereas the more tolerant M4 showed better maintenance of structural and metabolic functions. A flavanone 3-hydroxylase decreased in both genotypes, indicating that salt stress also affected flavonoid metabolism. Among defense-related proteins, chitinase class I basic increased in both rootstocks, while 101.14 showed marked accumulation of germin-like proteins and even induction of a bifunctional nitrilase/nitrile hydratase NIT4B-like protein, consistent with a more stressed physiological state. The same study also notes changes in glutathione transferase isoforms and a decrease in GDP-mannose 3,5-epimerase in 101.14, which may further compromise ascorbate-related antioxidant capacity. Overall, salt proteomics supports the view that M4 tolerance depends on sustaining ATP and NAD(P)H supply, vacuolar sequestration capacity, and osmoprotective metabolism, whereas 101.14 exhibits a root proteome typical of a more severely damaged tissue [104].

Nitrogen-related root proteomics is even less represented than abiotic-stress proteomics. Prinsi et al. [105] explicitly note that, at the time of publication, no proteomic investigation had yet examined nitrate responses in grapevine roots, which underlines how narrow the current evidence base still is. In their study of M4 roots, nitrate resupply after nitrogen starvation induced progressive nitrate accumulation, increased nitrate reductase abundance, and, after 30 h, increased amino acid levels accompanied by decreased reducing sugars and sucrose, indicating activation of nitrate assimilation at the expense of carbon reserves. Proteomically, this transition was characterized by increased nitrogen-assimilatory metabolism together with stimulation of the oxidative pentose phosphate pathway and glycolysis, interpreted as necessary to provide reducing power and carbon skeletons for assimilation. The authors also report broad modulation of protein and amino acid metabolism and changes in proteins involved in root development, emphasizing that nitrate availability affects not only assimilation enzymes such as nitrate reductase, glutamine synthetase, and glutamate synthase conceptually, but also the wider metabolic framework required to sustain nitrogen acquisition in root tissues. Their discussion is also consistent with a role for plasma membrane H^+^-ATPase and inducible nitrate uptake systems in linking external nitrate perception to metabolic reprogramming [105].

Cold-related root proteomics was addressed later by Chen et al. [107], who compared roots of a cold-resistant *Vitis riparia* × *V. labrusca* genotype with the cold-sensitive Cabernet Sauvignon. They explicitly state that differential protein expression in grapevine roots under freezing temperatures had not previously been identified, again emphasizing the limited representation of root proteomics within the broader grapevine field. Using iTRAQ-based analysis, they detected 36 and 57 differentially expressed proteins in the within-hybrid temperature comparison and in the hybrid-versus-Cabernet Sauvignon comparison, respectively, with 25 proteins co-expressed across treatments. These proteins were assigned to stress response, biosignaling, metabolism, energy, and translation, while root activity measurements confirmed the greater cold resistance of the interspecific hybrid. Although this study is less protein-rich in the main text than the Prinsi papers, it still supports the same broad conclusion emerging from the other root datasets: tolerant grapevine roots preserve activity through a coordinated proteomic response involving stress protection, biosignaling, and core metabolism, whereas sensitive roots undergo a sharper decline in functional capacity under stress [107].

Taken together, the currently available literature shows that grapevine root proteomics is still a niche and highly genotype-dependent area, dominated by a few systematic studies rather than a broad organ-level knowledge base. Even so, several recurring proteins and pathways emerge across the available experiments. These include antioxidant and detoxification proteins such as superoxide dismutase, ascorbate peroxidase, glutathione S-transferases, catalase, peroxiredoxins, and germin-like proteins; stress-protective proteins such as HSP70, HSP90, LEA-like proteins, chitinase class I, and chaperonins; metabolic enzymes such as phosphoglycerate kinase, glyceraldehyde-3-phosphate dehydrogenase, fructose-bisphosphate aldolase, malate dehydrogenase, pyruvate kinase, succinate dehydrogenase, pyruvate decarboxylase, alcohol dehydrogenase, and NADP-dependent malic enzyme; transport-related components such as NHX1, vacuolar H^+^-ATPase subunit E, and H^+^-PPase; and, under nitrate resupply, nitrate-assimilation-associated machinery centered on nitrate reductase and the broader glycolytic/oxidative pentose phosphate support network. What varies among genotypes is not the existence of these functional modules, but the extent to which they are maintained, amplified, or allowed to collapse under stress. This reinforces the conclusion that grapevine root resilience depends less on one single marker and more on the coordinated preservation of energetic competence, vacuolar ion handling, redox control, and protein homeostasis. Together, these data highlight the central role of the root proteome in orchestrating physiological resilience through metabolic flexibility, redox signaling, and structural adaptation (Table 4).

## 7. Cell Culture Proteomics

Cell suspension and callus cultures of *V. vinifera* have long served as simplified yet powerful models to study stress responses, secondary metabolism, and somatic embryogenesis under controlled conditions. Proteomic investigations of these systems have provided unique insights into defense-related pathways, signal transduction, and metabolite biosynthesis that would be difficult to dissect in intact plants.

Early comparative studies between embryogenic and non-embryogenic calli of Cabernet Sauvignon revealed that the acquisition of embryogenic competence is associated with a profound remodeling of the stress proteome, with enhanced accumulation of heat shock proteins (HSP70, HSP90), superoxide dismutase, and pathogenesis-related (PR) proteins, alongside enzymes of carbohydrate metabolism such as glyceraldehyde-3-phosphate dehydrogenase (GAPDH) and enolase [108]. Later, *Agrobacterium tumefaciens* transformation experiments using the same cultivar identified changes in proteins involved in oxidative stress control (ascorbate peroxidase, catalase), signal transduction (14-3-3 proteins, protein kinases), and protein folding (chaperonins), confirming that infection and transformation processes elicit defense-related pathways typical of plant–microbe interactions [109].

Elicitation studies using Gamay cell suspensions demonstrated that extracellular and intracellular proteomes undergo distinct remodeling upon exposure to various biotic and abiotic stimuli. Application of fungal elicitors led to the secretion of β-1,3-glucanases, chitinases, PR-5 thaumatin-like proteins, and peroxidases, reflecting the activation of apoplastic defenses. When challenged with methyl-β-cyclodextrin and methyl jasmonate, these cell cultures exhibited a massive production of stilbene synthase. This increase was accompanied by a higher abundance of enzymes involved in phenylpropanoid and oxidative metabolism, including phenylalanine ammonia-lyase, peroxidases, and superoxide dismutase. These observed proteomic changes are consistent with the accumulation of high levels of resveratrol, highlighting how these elicitors can reprogram the cell culture proteome to enhance the biosynthesis of defense compounds [110,111].

When challenged with methyl-β-cyclodextrin (MBCD) and methyl jasmonate, massive production of stilbene synthase was observed together with increased abundance of enzymes involved in phenylpropanoid and oxidative metabolism such as phenylalanine ammonia-lyase (PAL), peroxidases, and superoxide dismutase, consistent with high levels of resveratrol accumulation [110]. Similarly, treatment with chitosan induced alterations in defense and signaling proteins, further enhancing the synthesis and compartmentalization of stilbenes [112]. Studies on jasmonate signaling confirmed that jasmonic acid and Na-orthovanadate synergistically stimulate resveratrol biosynthesis, mediated by upregulation of stilbene synthase, PAL, and oxidative enzymes [113].

Proteomic analyses of cell cultures infected with *Botrytis cinerea* showed activation of pathogenesis-related proteins, protease inhibitors, and enzymes involved in ROS detoxification and amino acid metabolism, indicating that cultured cells can mount defense responses resembling those of intact leaves [114].

Temperature-dependent changes have also been extensively investigated. Quantitative proteomics on Cabernet Sauvignon cells exposed to heat and cold stress demonstrated dynamic modulation of sugar metabolism enzymes (sucrose synthase, fructose-bisphosphate aldolase), proteins of the phenylpropanoid pathway, and redox regulators (thioredoxin, glutathione S-transferase), suggesting that both metabolic flexibility and antioxidant capacity determine thermal tolerance [115]. Complementary work on grape berry–derived cell suspensions indicated that cultured cells reproduce key features of berry ripening, including changes in cell wall metabolism, lipid metabolism, and secondary metabolite biosynthesis, validating them as suitable systems for developmental studies [116].

Proteomic studies have also contributed to understanding somatic embryogenesis in *V. vinifera*. Quantitative analysis revealed differential abundance of lipid transfer proteins (LTPs), peroxidases, and embryogenesis-related chaperones, as well as enzymes involved in energy metabolism, underpinning the transition from undifferentiated to embryogenic states [117].

Collectively, proteomic analyses of grapevine cell cultures reveal that the in vitro environment, while simplified, recapitulates core defense and stress signaling networks observed in planta. Moreover, these systems provide valuable platforms for the discovery of regulatory proteins controlling secondary metabolism and developmental plasticity in grapevine (Table 5).

## 8. Grape Berry Proteomics

Comparative proteomics consistently shows that grape berries carry cultivar-specific protein fingerprints reflecting primary and secondary metabolism, stress preparedness, and cell-wall dynamics.

Using high-resolution mass spectrometry, Carpentieri et al. profiled white cultivars Fiano, Greco and Falanghina and demonstrated that an MS-based “molecular fingerprint” can reliably discriminate these Campanian varieties. The proteomic space resolved cultivar clusters and highlighted marker proteins that together encode varietal identity, supporting proteomics as a practical tool for authentication and typicity assessment [118]. A data-independent acquisition (DIA) survey of mature berries from the German red cultivar Dornfelder quantified ~700 proteins with absolute amounts estimated by TOP3, revealing that a large fraction mapped to stress/defense functions, while all glycolytic key enzymes were detected, indicating that glycolysis remains active at technological maturity. Enzymes of central carbon metabolism dominated together with redox-related proteins, whereas malate-based gluconeogenesis appeared to be of minor relevance at this stage [119].

Cross-genotype comparison of berry skins from red grapes with varying hybrid character (pure *V. vinifera* vs. interspecific hybrids with *V. labrusca* ancestry) using bottom-up label-free shotgun proteomics showed clear proteome-wide differentiation. Differentially abundant proteins concentrated in flavonoid/phenylpropanoid pathways, oxidative/ROS control, pathogenesis-related proteins, and cell-wall remodeling, matching divergent pigment and aroma traits across biotypes [120].

Finally, an integrated five-omics analysis of berry skins from five wine cultivars (Cabernet Sauvignon, Merlot, Pinot Noir, Chardonnay, Semillon) demonstrated that transcript, protein, and metabolite layers independently recapitulate cultivar separation, with highly concordant PCA structures across platforms. Notably, global protein–transcript correlations were modest at the gene level, underscoring post-transcriptional regulation; nevertheless, functional categories distinguishing cultivars converged on primary carbon metabolism, secondary metabolism (flavonoids/stilbenes), stress responses, and cell-wall processes [121].

Taken together, these studies establish that berry proteomes encode robust cultivar signatures. Core features include (i) preserved central carbon flux (glycolysis) at maturity, (ii) differential investment in phenylpropanoid/anthocyanin machinery in colored cultivars and hybrids, (iii) constitutive stress/PR protein baselines that vary by genotype, and (iv) cell-wall protein sets linked to texture and ripening. Multi-omics concordance at the pathway level, but weak mRNA–protein coupling gene-by-gene, highlights the value of direct proteome readouts for varietal characterization.

### 8.1. Proteomics of Grape Berry Ripening

Grape berry ripening represents a complex developmental transition involving coordinated physiological and biochemical changes that determine fruit composition, quality, and sensory attributes. Proteomic analyses across multiple *Vitis vinifera* cultivars and hybrid species have provided significant insights into how metabolic networks are regulated during this process.

Early proteomic investigations using 2-DE and MALDI-TOF revealed a global decrease in proteins associated with photosynthesis, carbon fixation, and energy metabolism, coupled with an increase in proteins linked to defense, stress adaptation, and secondary metabolism. In the Cabernet Sauvignon and Syrah cultivars, Giribaldi et al. identified a decline in RuBisCO, oxygen-evolving enhancer proteins, and ATP synthase subunits as ripening progressed, highlighting the transition from autotrophy to heterotrophy [122].

Quantitative DIGE-based proteomics further demonstrated that proteins involved in glycolysis (enolase, glyceraldehyde-3-phosphate dehydrogenase), the TCA cycle (malate dehydrogenase, citrate synthase), and sugar transport were upregulated in berry flesh during the mid- to late-ripening stages. This shift was accompanied by a coordinated decrease in enzymes of organic acid metabolism, including malic enzyme and phosphoenolpyruvate carboxylase, indicating organic acid consumption as sugars accumulate [123]. Label-based quantitative proteomics using iTRAQ expanded this view, identifying hundreds of differentially expressed proteins across developmental stages. Martínez-Esteso et al. demonstrated the reprogramming of central carbon metabolism, with strong upregulation of glycolytic and vacuolar transport proteins, suggesting enhanced sugar import and storage [124,125]. Proteins associated with vesicular trafficking (V-ATPase subunits, tonoplast intrinsic proteins) and stress responses (heat shock proteins, ascorbate peroxidase) were also significantly enriched during late ripening [125].

Omics integration further clarified the connections between proteomic and metabolomic profiles. Zamboni et al. used systems-level approaches to correlate proteome shifts with metabolite accumulation, revealing that enzymes of the phenylpropanoid and flavonoid pathways—such as chalcone synthase, stilbene synthase, and phenylalanine ammonia-lyase—exhibited cultivar-specific dynamics during veraison and ripening [126]. Similar integrative analyses linked oxidative stress-related enzymes (peroxidases, superoxide dismutases, and glutathione-S-transferases) to anthocyanin accumulation and defense priming in berry skins [127].

Comparative proteomics between red and white cultivars underscored pigmentation-related divergence, as proteins involved in anthocyanin biosynthesis (chalcone isomerase, flavanone 3-hydroxylase, UDP-glucose flavonoid glucosyltransferase) were selectively induced in red cultivars during fruit coloration [128]. Moreover, vacuolar transporters and glycosyltransferases were key in maintaining anthocyanin stability and compartmentalization [129].

The Pinot Noir skin proteome exhibited pronounced activation of proteins linked to stress and defense, including pathogenesis-related proteins, glutathione peroxidase, and chitinases, confirming that late ripening is accompanied by stress-like signaling [130]. Proteomic analyses of American hybrid cultivars have provided a broader perspective on how metabolic specialization contributes to varietal aroma and flavor development. In *V. rotundifolia* cv. ‘Carlos’ and the interspecific hybrid ‘Blanc du Bois’, both iTRAQ-based quantitative proteomics and LC–MS/MS analyses revealed extensive proteome remodeling throughout berry development and ripening [131,132,133]. During mid-ripening, there was a marked upregulation of proteins associated with amino acid metabolism, including S-adenosylmethionine synthase (SAMS), aspartate aminotransferase, and glutamate dehydrogenase, which play crucial roles in the generation of amino acid–derived aroma precursors. Parallel enhancement of enzymes involved in lipid metabolism, notably lipoxygenases (LOX) and alcohol dehydrogenases (ADH), suggested an increased flux through the lipoxygenase pathway, leading to higher production of C6 aldehydes and alcohols—volatile compounds contributing to the characteristic green and fruity aromas of Muscadine and hybrid grapes. Proteins related to the methionine and ethylene biosynthetic pathways, including 1-aminocyclopropane-1-carboxylate oxidase (ACO) and ethylene-forming enzymes, were also detected in higher abundance in late ripening stages, pointing to a potential role of ethylene signaling in the regulation of flavor metabolism and cell wall softening [132,133]. Additionally, differential expression of transport proteins, such as vacuolar H^+^-ATPases and ABC transporters, supported the mobilization of secondary metabolites, particularly phenolics and aroma-related precursors, into storage compartments. Overall, these studies demonstrated that the proteomic landscape of hybrid cultivars is characterized by enhanced activity of amino acid, lipid, and secondary metabolic enzymes, integrating flavor formation with stress-responsive pathways. This tight metabolic coordination underlies the distinctive aromatic complexity and resilience of hybrid grape berries grown in warmer or more humid environments typical of southeastern United States viticulture [129,130,131].

In addition, metabolomic–proteomic coupling in Syrah and Cabernet Sauvignon cultivated in warm climates revealed differential accumulation of heat shock proteins, redox enzymes, and phenylpropanoid-related proteins, reflecting adaptive metabolic responses to high temperature during late ripening [134]. The integration of predicted protein databases derived from expressed sequence tag (EST) collections has significantly improved the depth and reliability of proteomic identification in grape berries. Earlier proteomic analyses had been limited by the incomplete annotation of the *Vitis vinifera* genome, resulting in many unidentified or misassigned peptides. To overcome this, Lücker et al. [135] generated a comprehensive predicted protein database from large-scale EST data of Cabernet Sauvignon, which provided a refined reference for subsequent iTRAQ and LC–MS/MS analyses. This approach markedly enhanced the detection of low-abundance regulatory proteins, including transcription factors, signaling components, and enzymes of secondary metabolic pathways that had previously escaped identification using standard grape proteome databases. Notably, the integration of the EST-derived dataset enabled more confident mapping of vacuolar transporters, membrane-bound enzymes, and stress-related isoforms of proteins involved in phenolic metabolism, such as stilbene synthases and peroxidases. Beyond improving annotation accuracy, this development expanded the analytical scope of grape berry proteomics, facilitating comparative and quantitative studies across cultivars and ripening stages. It laid the foundation for integrative omics approaches in *Vitis* research, bridging transcriptomic and proteomic datasets to uncover regulatory networks that control sugar partitioning, phenylpropanoid biosynthesis, and cell wall modification during berry maturation [133].

Collectively, these proteomic investigations reveal that grape berry ripening is orchestrated by a comprehensive metabolic reprogramming that reshapes nearly every major biochemical pathway. The shift from a photosynthetically active to a metabolically specialized organ is accompanied by the progressive downregulation of photosynthetic and Calvin cycle proteins, including RuBisCO subunits, oxygen-evolving enhancer proteins, and chlorophyll-binding complexes [121,122]. This decline coincides with a pronounced increase in enzymes of sugar metabolism, particularly those involved in glycolysis and sucrose catabolism, such as fructose-bisphosphate aldolase, enolase, and glyceraldehyde-3-phosphate dehydrogenase, which sustain the energetic and biosynthetic demands of ripening [2,3,4]. In parallel, proteins of the tricarboxylic acid (TCA) cycle and associated respiratory pathways—such as malate dehydrogenase, aconitase, and citrate synthase—exhibit increased abundance during veraison, reflecting a strong metabolic rerouting toward energy generation and organic acid turnover [123,126]. These changes contribute to the depletion of malic acid and the accumulation of sugars characteristic of the ripening process. Secondary metabolism is also extensively remodeled, as evidenced by the upregulation of phenylpropanoid pathway enzymes, including phenylalanine ammonia-lyase, chalcone synthase, stilbene synthase, and flavanone-3-hydroxylase, which drive the biosynthesis of flavonoids, tannins, and stilbenes [126,127,128]. These metabolites not only determine berry color, aroma, and astringency but also enhance protection against oxidative stress and pathogen attack. The concurrent induction of pathogenesis-related proteins (PRs), peroxidases, and glutathione-S-transferases supports the notion that late ripening stages are associated with oxidative and defense priming, a molecular readiness to counter environmental or biotic stressors [130]. Stress- and signaling-related proteins—such as heat shock proteins (HSP70, HSP90), aspartate aminotransferase, and superoxide dismutase—are increasingly abundant in late ripening berries [127,131,134]. Their accumulation suggests an adaptive stress response that stabilizes protein conformation and preserves redox homeostasis under the combined influence of dehydration, high temperature, and metabolic activity. These protective systems are particularly enhanced in cultivars grown in warm or arid regions, where proteomic signatures indicate increased antioxidant capacity and metabolic plasticity [134,136]. Another hallmark of ripening is the expansion of the vacuolar transport and membrane trafficking machinery. Enhanced expression of V-ATPase subunits, tonoplast intrinsic proteins (TIPs), and ABC transporters facilitates the storage and compartmentalization of sugars, organic acids, anthocyanins, and aromatic precursors [125,129,135]. This vacuolar specialization is tightly integrated with energy metabolism and secondary biosynthesis, ensuring efficient metabolite sequestration and homeostasis during rapid cellular expansion.

Recent advances in integrative omics approaches have provided new insight into the metabolic regulation of grape berries under overripening conditions. Shi et al. [137] performed a comprehensive multi-omics study combining proteomic, transcriptomic, and metabolomic analyses of Cabernet Sauvignon. Using an iTRAQ-based quantitative LC–MS/MS workflow, the authors identified 1270 proteins, among which 197 were consistently differentially expressed in both vintages. The results revealed a pronounced metabolic reprogramming as berries transitioned beyond physiological maturity. Proteins associated with primary metabolism, including enzymes of sugar, fatty acid, and amino acid biosynthesis, were generally downregulated, indicating a decline in anabolic capacity during the late stages of ripening. In contrast, proteins related to protein processing, degradation, and stress responses—notably heat shock proteins (HSP70, HSP90), small HSPs, Rab GTPases, aquaporins, and other chaperones—were markedly upregulated, reflecting enhanced proteostasis and cellular protection under dehydration and temperature stress. Additionally, enzymes linked to secondary metabolism (stilbene synthase, phenylalanine ammonia-lyase, and isoflavone reductase) showed decreased abundance, suggesting a shift from phenolic biosynthesis toward energy conservation and senescence pathways. Integration with transcriptomic and metabolomic data demonstrated strong year-to-year variability in the intensity of these changes, highlighting the influence of environmental stressors—particularly water deficit and temperature fluctuations—on late-ripening metabolic plasticity. Overripe grape berries adapt by reallocating metabolic resources from growth and biosynthesis to stress tolerance, osmotic adjustment, and cellular maintenance.

**Figure 11:**
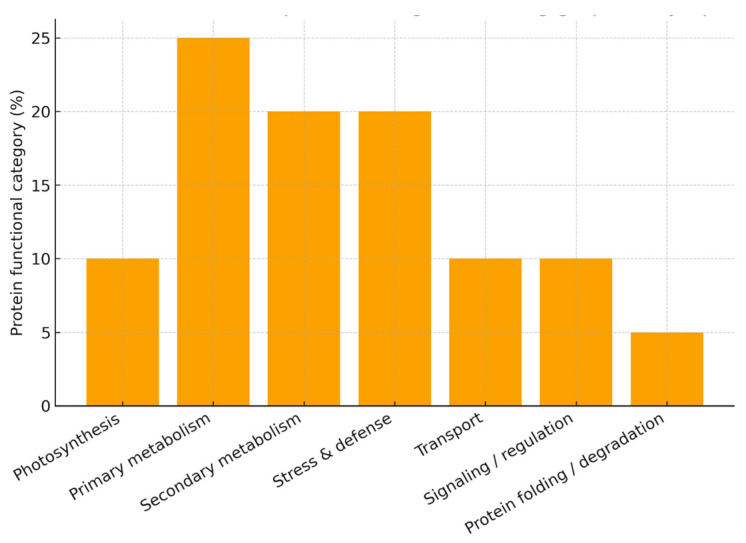
Distribution of functional protein categories during grape berry ripening. Values represent the relative proportion of proteins assigned to each category, normalized to 100% for the dataset. These values reflect the distribution of functional categories rather than absolute protein abundance. Data were compiled from published proteomic studies.

Taken together, these findings demonstrate that grape berry ripening represents a highly coordinated proteome transition, integrating carbohydrate metabolism, redox regulation, and secondary metabolite production (Figure 11, Table 6). The dynamic interplay between primary and secondary metabolism, stress adaptation, and transport processes defines the biochemical identity of each cultivar and ultimately determines wine quality and terroir expression. Modern quantitative proteomics—particularly label-based approaches such as iTRAQ and DIA—has proven indispensable for unraveling these intricate molecular events, providing a holistic framework for understanding fruit maturation at the systems level [123,124,129,132,133,135].

### 8.2. Berry Proteome Under Abiotic Factors

Proteomic analyses of grape berries under abiotic stress conditions—including high temperature, water deficit, light exposure, and hormonal treatments—have provided important insights into the adaptive metabolic responses that shape fruit composition and quality.

In a foundational study, Grimplet et al. [138] performed a comparative proteomic and metabolomic analysis of Cabernet Sauvignon berry tissues (skin, pulp, and seeds) under well-watered and water-deficit conditions. Approximately 7% of proteins were found to be differentially expressed in response to drought. The skin proteome showed increased abundance of pathogenesis-related (PR) proteins, proteasome components, antioxidant enzymes, and flavonoid biosynthetic enzymes, while the pulp displayed upregulation of primary metabolic enzymes and glutamate decarboxylase, suggesting a coordinated adjustment in osmotic balance and redox homeostasis.

The impact of elevated temperature on berry development was explored by Lecourieux et al. [139] using a combined label-free quantitative proteomics and metabolomics approach. Out of 2279 identified proteins, 592 were differentially abundant in heat-exposed berries. High temperature disrupted carbohydrate and energy metabolism, leading to a reduction in photosynthetic and glycolytic enzymes, while antioxidant defenses, chaperones, and heat shock–related signaling proteins were markedly induced. The authors also reported a clear decoupling between transcriptomic and proteomic responses, highlighting the role of post-transcriptional regulation in thermal adaptation.

In another integrative study, Olmedo et al. [140] investigated the effects of pre-flowering cytokinin (CPPU) applications on the central carbon metabolism of Thompson Seedless berries. Proteomic and metabolomic integration revealed an enhancement of glycolytic and tricarboxylic acid (TCA) cycle enzymes, accompanied by higher levels of glucose, fructose, and sucrose, pointing to a cytokinin-driven reprogramming of sugar and organic acid metabolism that contributes to berry growth and ripening.

The effect of light exposure was addressed by Teixeira et al. [141], who demonstrated that increased light intensity stimulates light reactions but inhibits the Calvin cycle in berry skin chloroplasts of red grape cultivars. Enhanced abundance of photosystem II proteins, electron transport components, and antioxidative enzymes suggests an acclimatory response aimed at maintaining photochemical balance and protecting against photooxidative stress, while the suppression of Calvin cycle enzymes indicates a metabolic shift toward photoprotection rather than carbon assimilation.

At a systems level, Serrano et al. [142] integrated transcriptomic, metabolomic, and proteomic data to construct regulatory networks describing grapevine responses to environmental stimuli. The study showed that water deficit, high temperature, and UV exposure collectively induce the accumulation of anthocyanins, flavonols, and stilbenes, as well as stress-related proteins involved in ROS detoxification and secondary metabolism, thereby reinforcing berry resilience under challenging conditions.

Taken together, these studies reveal that abiotic stresses lead to differential regulation of proteins involved in energy metabolism, oxidative stress defense, phenolic biosynthesis, and osmoprotection (Table 7). The magnitude and direction of these changes depend on both the type of stress and the developmental stage of the berry. Moreover, the observed discrepancies between transcript and protein levels underscore the critical importance of post-transcriptional and translational mechanisms in fine-tuning berry stress responses. Integrating proteomic data with metabolomic and transcriptomic analyses provides a comprehensive understanding of how environmental conditions shape grape berry metabolic plasticity and, ultimately, its technological and sensory quality.

### 8.3. Berry Proteome Under Biotic Stress

Proteomic studies investigating biotic stress in grape berries have revealed complex molecular responses encompassing defense activation, redox control, structural reorganization, and secondary metabolism adjustments. These changes are often tissue-specific, particularly affecting the exocarp where the berry interfaces with external pathogens and environmental cues.

The study by Pasquier et al. [143] examined the impact of *Esca proper* symptoms on the protein composition of grape berry skins (Cabernet Sauvignon) using 2-DE followed by mass spectrometry. Thirteen proteins were identified as differentially expressed between healthy and symptomatic vines. These included defense-related and stress-associated proteins such as PR-5 thaumatin-like proteins, PR-10, polyphenol oxidase, and small heat shock proteins (sHSPs), along with enzymes involved in oxidative phosphorylation (ATP synthase, inorganic pyrophosphatase), redox regulation (aldehyde dehydrogenase, cysteine synthase, methionine sulfoxide reductase), and vitamin B6 biosynthesis. The results indicated that *Esca proper* induces an imbalance in the redox system and triggers both mitochondrial and chloroplastic oxidative stress responses, reflecting a dual adjustment to energy metabolism and defense signaling during ripening. Such modifications may alter sulfur-containing amino acid metabolism and consequently influence oenological properties of the fruit.

A complementary study by Giribaldi et al. [144] investigated Nebbiolo infected with phloem-limited viruses (Grapevine leafroll-associated virus 1 (GLRaV-1), Grapevine virus A (GVA), and Rupestris stem pitting-associated virus (RSPaV)) and combined agronomic, chemical, and proteomic analyses. Although virus infection caused limited agronomic impact, infected berries displayed higher titratable acidity and elevated resveratrol levels, suggesting stress-related activation of secondary metabolism. Proteomic profiling revealed induction of oxidative stress–related proteins such as polyphenol oxidase in skins, and modification of cytoskeleton- and signaling-associated proteins including α-tubulin, fimbrin, and Rab11 GTPase in pulp. These responses suggest that virus infection reorganizes vesicle trafficking and cellular architecture, potentially influencing berry texture and phenolic compound partitioning during ripening.

In berries affected by *Botrytis cinerea* (“noble rot”), Lorenzini et al. [145] identified pathogen-derived proteins accumulated in infected Corvina berries during the appassimento process for Amarone wine. Using a 2-DE/MS approach, the study detected peroxiredoxin-5, malate dehydrogenase, ATP synthase α and β subunits, hydroxysteroid dehydrogenase, acid protease, and several hypothetical fungal proteins (including the Woronin body major protein) as potential biomarkers of infection. Host proteins associated with antioxidant activity and phenolic metabolism were also altered, reflecting a strong redox component in the host–pathogen interplay. These protein markers hold potential for mass spectrometry–based monitoring of noble rot during grape drying and vinification.

Finally, Melo-Braga et al. [146] provided a multilayered view of berry defense through a comprehensive post-translational modification (PTM) proteomic analysis of mesocarp and exocarp from Italia following *Lobesia botrana* infestation. The authors quantified changes in 899 proteins, mapped over 100 phosphorylation sites, and identified 323 N-glycosylation and 138 lysine acetylation sites, revealing a dynamic PTM-mediated regulation of the berry’s defense network. Functional enrichment showed strong modulation of membrane trafficking, cell wall metabolism, ROS detoxification, and signal transduction, emphasizing the role of PTMs as fine-tuning mechanisms coordinating metabolic and structural defense layers in grape berries.

Together, these proteomic studies demonstrate that biotic stresses—including fungal pathogens, viral infections, and insect herbivory—elicit both conserved and specific molecular signatures in grape berries (Table 7). Common hallmarks include the activation of antioxidant and defense-related pathways, remodeling of cell wall and cytoskeletal components, and the modulation of energy and secondary metabolism. The integration of classical proteomics with PTM-focused and metabolomic approaches highlights the plasticity of the berry proteome, underpinning its capacity to maintain homeostasis and quality under biological challenges.

### 8.4. Berry Tissue–Specific Proteomes

Proteomic investigations of *V. vinifera* berry tissues have revealed highly specialized metabolic landscapes in the exocarp, mesocarp, and seeds, reflecting the tissue-specific physiological roles during fruit development and ripening. These studies, based on two-dimensional electrophoresis (2-DE), LC–MS/MS, and MALDI–TOF–MS, have elucidated differential protein expression patterns associated with photosynthesis, cell wall remodeling, secondary metabolism, stress responses, and seed storage processes.

In the exocarp, Deytieux et al. [147] identified over 200 proteins in Cabernet Sauvignon skins across ripening stages using 2-DE and MALDI–TOF–MS. The most abundant categories included cell wall-modifying enzymes (expansins, pectin methylesterases, polygalacturonases), defense-related proteins such as chitinases, β-1,3-glucanases, and thaumatin-like proteins, and enzymes involved in phenylpropanoid and flavonoid biosynthesis. Their expression progressively increased during veraison, suggesting an active role of the exocarp in structural defense and phenolic compound accumulation. Negri et al. [148] further characterized proteomic shifts in Barbera grape skins, observing that proteins related to oxidative stress regulation (superoxide dismutase, peroxidases) and secondary metabolism accumulated toward full ripeness. Similarly, Niu et al. [149] demonstrated that sunlight exclusion dramatically altered the abundance of flavonoid biosynthetic enzymes (chalcone synthase, flavanone 3-hydroxylase, dihydroflavonol 4-reductase) and stress-responsive proteins, leading to a reduction in anthocyanin content and cuticle-associated proteins. Together, these studies reveal that exocarp proteome dynamics integrate photoprotection, pigmentation, and pathogen defense in response to both developmental and environmental cues.

The mesocarp proteome presents a distinct metabolic signature primarily linked to carbohydrate metabolism, vacuolar transport, and energy production. Sarry et al. [150] performed one of the earliest comprehensive analyses using 2-DE and LC–MS/MS, identifying more than 300 protein spots. Approximately 34% of the identified proteins were associated with energy metabolism (glycolysis, TCA cycle, mitochondrial electron transport), 19% with stress and defense responses, 13% with primary metabolism, and 11% with protein synthesis and turnover. The prevalence of enzymes such as malate dehydrogenase, enolase, and ATP synthase reflects the high respiratory activity of the pulp, which supports sugar accumulation during ripening. The simultaneous presence of heat shock proteins (HSP70, HSP90) and ascorbate peroxidase indicates mechanisms of oxidative protection accompanying metabolic intensification.

In the seeds, proteomic studies have delineated both varietal differentiation and biochemical specialization. Early work by Pesavento et al. [151] and Bertazzo et al. [152] used MALDI–MS profiling of grape seed proteins to distinguish *V. vinifera* cultivars based on characteristic *m/z* patterns, highlighting the taxonomic and genetic potential of seed proteome fingerprints. Subsequent studies provided molecular insight into protein composition and solubility fractions. Zhou et al. [153] identified four major water-soluble proteins—oxysterol-binding protein, ADP-glucose pyrophosphorylase, NADP-sorbitol-6-phosphate dehydrogenase, and UDP-glycosyltransferase—that are central to carbohydrate and lipid metabolism. Gazzola et al. [154] characterized the seed endosperm proteome using Osborne fractionation and LC–MS/MS, revealing that 11S and 7S globulin-like storage proteins dominated the albumin–globulin fraction (approximately 58% of total proteins). These storage globulins, composed of 19–44 kDa polypeptides, are critical for nitrogen reserve accumulation and germination. Collectively, seed proteomic data illustrate a dual metabolic–structural nature, combining active enzymatic networks with storage protein assemblies that underpin both seed maturation and varietal identity.

**Figure 12:**
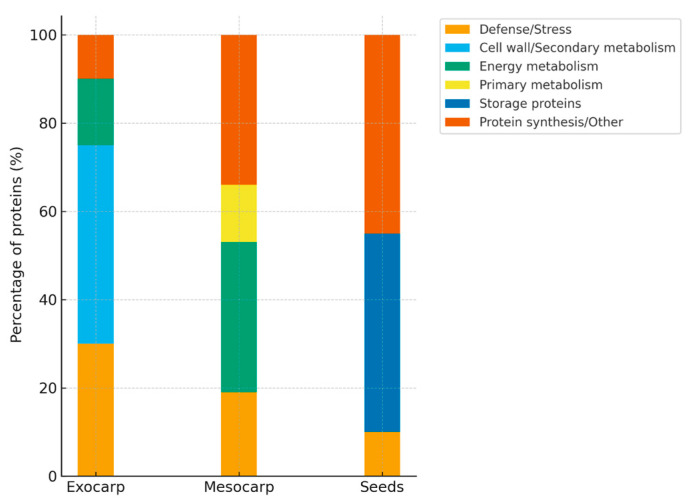
Distribution of proteins across *Vitis vinifera* berry tissues (exocarp, mesocarp, and seeds) based on published proteomic datasets. Values represent the relative proportion of proteins assigned to each functional category, normalized to 100% within each tissue. These values reflect the distribution of functional categories rather than absolute protein abundance.

Overall, tissue-specific proteomic investigations of grape berries have underscored the spatial compartmentalization of metabolic functions: the exocarp as a barrier and phenolic defense tissue; the mesocarp as a carbohydrate-rich energy center; and the seed as a reservoir of nitrogen and enzymes for early development (Figure 12). Together, these findings provide an integrated proteomic framework that connects the structural and metabolic specialization of berry tissues to fruit quality traits and developmental physiology.

### 8.5. Proteomic Characterization of Specific Proteins in Grape Berries

Targeted proteomic and biochemical studies have identified and functionally characterized several individual proteins or protein families that play pivotal roles in grape berry development, metabolism, and defense. Among them, miraculin-like proteins (MLPs), β-glucosidases, and pathogenesis-related (PR) proteins such as chitinases and β-1,3-glucanases have been the most extensively analyzed.

The miraculin-like protein from *V. vinifera* was purified and structurally characterized using X-ray crystallography and molecular modeling approaches, revealing a β-trefoil fold typical of the thaumatin family [155]. Biochemical assays confirmed that the grape MLP is localized predominantly in the skin and exhibits weak sweetness-modifying properties but strong antifungal activity. Its expression increases during ripening and in response to pathogen attack, suggesting a dual role in defense and fruit sensory modulation.

In a complementary study, β-glucosidase-enriched extracts from grape berries were analyzed by mass spectrometry and enzymatic assays, leading to the identification of multiple isoforms with distinct substrate specificities [156]. The predominant cytosolic isoform exhibited high affinity for monoterpenyl-glucosides, contributing to the release of volatile aroma compounds during winemaking. The proteomic mapping (MALDI-TOF combined with peptide sequencing) confirmed that these enzymes belong to the GH1 family, with conserved catalytic residues typical of retaining β-glucosidases.

A comparative proteomic analysis of Sauvignon Blanc berry tissues (skin, pulp, and seed) provided a quantitative overview of PR proteins across berry compartments [157]. Label-free LC–MS/MS revealed the differential distribution of PR-2 (β-1,3-glucanases), PR-3 and PR-4 (chitinases), PR-5 (thaumatin-like proteins), and PR-10 (pathogenesis-related proteins of unknown function). The skin contained the highest abundance of PR-10 and TLPs, while β-1,3-glucanases were predominant in seeds. This tissue-specific accumulation reflects functional specialization, where outer tissues provide a biochemical barrier against fungal invasion.

Detailed characterization of a Class IV chitinase from grape confirmed its structural and functional features [158]. The enzyme displayed a modular organization with an N-terminal signal peptide and a C-terminal cysteine-rich domain, enabling antifungal activity against *B. cinerea*. Expression profiling showed that Class IV chitinase transcripts accumulate in ripening berries and are strongly induced by biotic stress, linking their proteomic presence to both developmental and defensive functions.

Similarly, β-1,3-glucanase was purified and analyzed across grape berry tissues, confirming isoform-specific expression during fruit maturation [159]. Immunoblotting and proteomic identification demonstrated that the protein increases in the skin during veraison and in the seed coat at late stages of ripening. These enzymes are likely involved in remodeling the cell wall and mobilizing carbohydrate reserves, in addition to their role in pathogen defense.

Together, these studies highlight the integrative role of targeted proteomics in elucidating the biological significance of specific protein families in grape berry physiology. Beyond general defense functions, these proteins contribute to fruit quality traits including aroma development, texture, and pathogen resistance.

### 8.6. Integrative Mechanistic Framework of Grape Berry Proteome

Proteomic analyses across grape berry development, environmental conditions, and biotic interactions consistently reveal that the berry proteome operates as a highly coordinated and dynamically reprogrammed system, integrating primary metabolism, secondary metabolite biosynthesis, stress responses, and intracellular transport processes. Despite the diversity of developmental stages, genotypes, and external stimuli, these responses converge on a limited number of core functional modules that define berry physiology and quality [118,119,120,121,123,124,125,126,142].

A central feature of berry proteome dynamics is the progressive metabolic transition from a photosynthetically active tissue to a heterotrophic, storage-driven organ. This shift is characterized by the downregulation of photosynthetic proteins, including RuBisCO subunits and chlorophyll-associated complexes, accompanied by sustained or increased activity of glycolysis and the tricarboxylic acid (TCA) cycle. This metabolic reconfiguration ensures continuous energy supply and carbon flux required for sugar accumulation, organic acid turnover, and biosynthetic processes during ripening [122,123,124,125,126].

In parallel, secondary metabolism becomes increasingly dominant, particularly through activation of phenylpropanoid and flavonoid pathways. Enzymes such as phenylalanine ammonia-lyase, chalcone synthase, and stilbene synthase are differentially regulated depending on genotype, tissue, and environmental conditions, linking proteome composition to pigmentation, aroma formation, and defense capacity. This metabolic branch is tightly coupled with redox regulation, as accumulation of flavonoids and stilbenes contributes to oxidative stress mitigation and pathogen resistance [120,126,127,128,134].

Redox homeostasis and stress-related signaling represent another conserved layer of proteome organization. Across developmental stages, abiotic stress conditions, and pathogen interactions, consistent induction of antioxidant enzymes (superoxide dismutase, peroxidases, glutathione S-transferases) and molecular chaperones (HSP70, HSP90) reflects the necessity to buffer oxidative stress arising from high metabolic activity, dehydration, and environmental fluctuations. This stress-associated proteomic signature becomes particularly pronounced during late ripening and overripening, where metabolic activity and environmental constraints converge [125,127,130,134,137].

Efficient compartmentalization and transport processes further define berry proteome functionality. The coordinated upregulation of vacuolar transporters (V-ATPases, tonoplast intrinsic proteins, ABC transporters) enables sequestration of sugars, organic acids, phenolics, and aroma precursors into cellular compartments, ensuring both metabolic homeostasis and the development of characteristic fruit composition. This transport network is closely integrated with primary and secondary metabolism, forming a functional axis that supports rapid cellular expansion and biochemical specialization [125,129,135].

At the structural level, tissue-specific proteomes introduce an additional layer of organization. The exocarp is enriched in defense-related and phenylpropanoid enzymes, functioning as a protective interface, while the mesocarp is dominated by energy metabolism and sugar accumulation processes. Seeds, in contrast, combine active metabolic pathways with storage protein assemblies, reflecting their dual role in nutrient storage and future germination. This spatial compartmentalization ensures that metabolic functions are distributed according to physiological requirements within the berry [147,148,149,150,151,152,153,154,157].

A critical determinant of berry proteome behavior is the extensive role of post-transcriptional and post-translational regulation. The relatively weak correlation between transcript and protein abundance, together with widespread phosphorylation, acetylation, and glycosylation events, indicates that proteome composition is largely controlled beyond transcription. These regulatory layers enable rapid and context-dependent adjustment of metabolic and defense pathways, particularly under fluctuating environmental conditions and during stress responses [121,146].

Collectively, these findings define a unified mechanistic framework in which grape berry development and stress adaptation are governed by the integration of four interconnected processes: (i) reprogramming of primary carbon metabolism to sustain energy and biosynthetic demands; (ii) activation of secondary metabolic pathways that determine quality traits and defense capacity; (iii) maintenance of redox and proteostasis networks to stabilize cellular function; and (iv) spatial and subcellular compartmentalization of metabolites and proteins. The coordination and timing of these processes ultimately determine berry composition, resilience, and varietal typicity, providing a systems-level understanding of how proteome dynamics translate into fruit quality and technological potential [118,119,120,121,123,124,125,126,134,142].

## 9. Wine Proteomics

The proteomic composition of wine reflects the selective survival of a limited group of grape-derived proteins that withstand the drastic chemical, enzymatic, and physical transformations occurring during vinification and aging. Importantly, the majority of proteins detected in wine originate directly from grape tissues and therefore represent the final, highly selective stage of plant proteome persistence under extreme physicochemical conditions. While thousands of proteins are present in the grape berry, only a small subset—primarily acidic, cysteine-rich, and structurally compact proteins—remains soluble and stable under the combined effects of low pH, ethanol, and oxidative conditions. Over the past two decades, proteomic approaches such as two-dimensional electrophoresis (2-DE) and LC–MS/MS have enabled detailed characterization of these proteins and their functional roles in wine [160].

Early chromatographic and proteomic studies demonstrated that the wine proteome represents a simplified subset of the grape proteome, dominated by proteins in the 15–35 kDa range with high resistance to proteolysis and denaturation [160]. Subsequent analyses identified these proteins mainly as pathogenesis-related (PR) proteins, including thaumatin-like proteins (TLPs) and class IV chitinases, together with lipid transfer proteins (LTPs), β-1,3-glucanases, peroxidases, and polygalacturonase-inhibiting proteins (PGIPs) [161,162]. These proteins are not randomly retained but reflect specific functional groups originally involved in plant defense, cell wall remodeling, and stress adaptation, indicating that the wine proteome retains key signatures of grapevine physiological responses.

The predominance of PR proteins in wine is directly linked to their structural properties. TLPs (PR-5 family) and chitinases (PR-3 family) are cysteine-rich proteins stabilized by multiple disulfide bridges, forming compact and highly ordered structures. TLPs typically exhibit a β-barrel fold stabilized by up to eight disulfide bonds, whereas chitinases contain an α-helical catalytic domain and a chitin-binding region [162,163,164,165]. These features confer exceptional resistance to acidic pH, ethanol, and proteolytic degradation, enabling their persistence throughout fermentation and storage.

Despite their intrinsic stability, these proteins are not completely inert. Under conditions such as elevated temperature, ethanol-induced conformational stress, or oxidative changes, TLPs and chitinases may undergo partial unfolding. This process exposes hydrophobic regions that promote protein–protein interactions and aggregation, leading to haze formation. Comparative studies have shown that chitinases are generally more prone to irreversible unfolding due to their more open tertiary structure and lower glycosylation, whereas TLPs are more structurally resilient but can still participate in aggregation under prolonged stress conditions [163,164].

Beyond TLPs and chitinases, the wine proteome includes a number of minor yet functionally significant proteins. Lipid transfer proteins (LTPs), typically around 9 kDa, are small, amphipathic, and highly stable molecules capable of binding hydrophobic compounds. In wine, they are associated with aroma compound interactions and contribute to foam stability, particularly in sparkling wines. Their structural stability is also linked to disulfide bond formation, similar to PR proteins, reinforcing the importance of cysteine-rich proteins in the wine proteome [160,166].

Polygalacturonase-inhibiting proteins (PGIPs) represent another important component, originally functioning in plant defense by inhibiting fungal enzymes involved in cell wall degradation. In wine, PGIPs may influence interactions between proteins and polysaccharides, thereby contributing to mouthfeel and colloidal stability. β-1,3-glucanases, although less abundant, can interact with polysaccharides and yeast-derived mannoproteins, affecting filtration properties and the structural organization of the wine matrix.

Peroxidases and other oxidoreductases present in wine contribute to oxidative reactions that influence color stability and redox balance. These enzymes, originally involved in plant stress responses, remain partially active or structurally relevant in wine, linking oxidative processes in the final product to their biological roles in the grape.

Proteomic and peptidomic studies have also revealed that partial proteolysis of PR proteins occurs during wine storage. Peptides derived from exposed regions of TLPs and chitinases have been identified, indicating limited cleavage at flexible or solvent-accessible sites [165]. These peptides may contribute to the dynamic equilibrium between soluble and aggregated protein forms, influencing haze formation and long-term stability.

The concentration and composition of proteins in wine are influenced by multiple factors, including grape variety, environmental conditions during ripening, grape health, and vinification practices. White wines typically contain higher concentrations of soluble proteins (10–100 mg/L), whereas in red wines, interactions with polyphenols promote protein precipitation or formation of stable complexes, contributing to improved colloidal stability and reduced haze formation [166,167].

Collectively, the wine proteome represents a chemically selective and functionally resilient subset of the grape proteome (Table 8). Dominated by pathogenesis-related proteins and complemented by LTPs, PGIPs, peroxidases, and glucanases, it provides a unique system for studying protein stability, structural resilience, and functional persistence beyond the cellular environment. In this context, wine proteomics extends plant proteomics into a post-harvest system, revealing how specific structural and biochemical properties enable certain plant proteins to retain functionality under extreme physicochemical conditions.

## 10. Post-Translational Modifications

Post-translational modifications (PTMs) represent a critical regulatory layer of the grapevine proteome, enabling rapid and reversible modulation of protein function in response to developmental and environmental cues. Although PTM-focused studies in *Vitis* remain relatively limited, available evidence demonstrates their key role in coordinating stress signaling, metabolism, and defense responses.

Phosphorylation is the most extensively characterized PTM in grapevine, particularly in leaf responses to abiotic stress and hormonal signaling. Phosphoproteomic analysis of *Vitis vinifera* leaves exposed to abscisic acid (ABA) revealed extensive remodeling of the phosphoproteome, with hundreds of phosphorylated proteins and numerous phosphorylation sites identified. Many of these proteins are involved in photosynthesis, primary metabolism, and protein synthesis, indicating that phosphorylation plays a central role in regulating energy balance and growth under stress conditions. In addition, ABA signaling was shown to operate through canonical kinase pathways, including SnRK2-mediated phosphorylation cascades, which modulate transcription factors and downstream stress-responsive proteins [97].

Complementary insights into stress-induced proteome regulation are provided by quantitative proteomic analyses of heat-stressed leaves. iTRAQ-based studies demonstrated large-scale changes in proteins associated with photosynthesis, antioxidant defense, and protein folding, including heat shock proteins and enzymes of redox metabolism. Although these studies primarily focus on protein abundance, they also emphasize that heat stress affects protein stability, folding, and turnover—processes that are tightly regulated by PTMs such as phosphorylation and acetylation [91].

In grape berries, PTMs have been investigated in the context of biotic stress using multi-parallel proteomic approaches. A comprehensive study of Lobesia botrana infection revealed simultaneous modulation of phosphorylation, N-glycosylation, and lysine acetylation in mesocarp and exocarp tissues. This analysis identified more than 1100 phosphorylation sites, over 300 glycosylation sites, and more than 100 acetylation sites, demonstrating the extensive scope of PTM-mediated regulation in grapevine tissues. Differentially modified proteins were associated with photosynthesis, metabolism, and defense pathways, while motif analysis indicated the activation of specific kinase-driven signaling networks [146].

Beyond direct PTM identification, integrative multi-omics studies further underscore the importance of post-transcriptional and post-translational regulation in grapevine biology. Comparative analyses of transcriptomic, proteomic, and metabolomic datasets revealed a generally low correlation between mRNA and protein abundance in berry tissues, highlighting the importance of regulatory mechanisms beyond transcription [121].

Collectively, these studies demonstrate that PTMs contribute to a dynamic and multilayered regulatory network in *Vitis*, enabling fine-tuning of metabolic processes, stress responses, and developmental transitions. However, compared to model plant systems, PTM-focused research in grapevine remains fragmented and largely restricted to specific conditions such as ABA signaling, heat stress, and pathogen interactions. Future research integrating phosphoproteomics, acetylproteomics, and glycoproteomics across organs, developmental stages, and environmental gradients will be essential for achieving a comprehensive understanding of PTM-mediated regulation in grapevine biology.

## 11. Conclusions and Future Perspectives

Proteomic research across the genus *Vitis* has evolved into a central framework for understanding grapevine biology, integrating molecular, physiological, and developmental dimensions. Advances in mass spectrometry–based proteomics, including high-resolution LC–MS/MS, label-free quantification, and data-independent acquisition (DIA), have enabled increasingly detailed characterization of protein composition, dynamics, and functional regulation across different grapevine organs and conditions.

Proteomic studies of grapevine organs have provided key insights into the complexity of plant responses to both biotic and abiotic stresses. In leaves, extensive datasets have revealed dynamic regulation of photosynthesis, redox homeostasis, and defense pathways in response to pathogens such as *Plasmopara viticola*, *Erysiphe necator*, Flavescence dorée, and *Xylella fastidiosa*, as well as environmental stressors including drought, salinity, temperature, and light. These responses are mediated by coordinated changes in primary metabolism, antioxidant systems, and pathogenesis-related proteins, reflecting the integration of stress perception with metabolic reprogramming.

Complementary insights have been obtained from less extensively studied organs. Bud proteomics has highlighted the molecular regulation of dormancy, including the involvement of stress-related proteins, redox regulation, and cytoskeletal remodeling in dormancy maintenance and release. Root proteomics, although limited in scope, has revealed key mechanisms of adaptation to drought, salinity, and nutrient availability, particularly through modulation of energy metabolism, ion transport, and oxidative stress responses.

Developmental proteomics has further expanded our understanding of grapevine biology, particularly in berries, where protein dynamics reflect the transition from fruit set to ripening. Proteomic analyses have identified coordinated changes in carbohydrate metabolism, vacuolar transport, organic acid metabolism, and stress-related proteins that underpin berry development and varietal characteristics. Tissue-specific studies have emphasized the spatial complexity of the berry proteome, with distinct functional specializations in the exocarp, mesocarp, and seeds.

Collectively, these studies highlight the grapevine proteome as a dynamic and multilayered system, in which conserved protein families—including heat shock proteins, antioxidant enzymes, and pathogenesis-related proteins—are differentially regulated across organs, developmental stages, and environmental conditions. The frequent occurrence of protein isoforms, together with diverse post-translational modifications such as phosphorylation, acetylation, and ubiquitination, further contributes to this complexity, underscoring the importance of context-dependent interpretation of proteomic data.

Looking ahead, the integration of proteomics within broader systems biology frameworks will be essential for advancing grapevine research. Future studies should increasingly focus on quantitative, spatially resolved, and temporally dynamic analyses that capture proteome changes at high resolution across tissues and developmental stages. Advances in single-cell proteomics, subcellular fractionation, and post-translational modification profiling will further refine our understanding of regulatory networks controlling metabolism, signaling, and stress responses.

A major opportunity lies in the integration of proteomics with transcriptomic, metabolomic, and phenotypic datasets, enabling the identification of robust molecular markers associated with stress resilience, development, and productivity. Such integrative approaches will support the development of predictive models linking environmental conditions to plant performance and will contribute to the advancement of precision viticulture and targeted breeding strategies.

An important emerging research gap concerns the impact of heavy metal stress on the grapevine proteome. Although individual studies have addressed the physiological effects of elements such as manganese, comprehensive proteomic investigations of heavy metal exposure in *Vitis* remain extremely limited. This is particularly relevant given the increasing incidence of soil contamination associated with industrial activity, intensive agriculture, and the long-term use of copper-based fungicides in vineyards. Future research should therefore focus on identifying metal-responsive proteins, detoxification mechanisms, and oxidative stress pathways, as well as on integrating proteomics with ionomics and metabolomics to better understand the effects of metal accumulation on grapevine physiology and fruit development. Addressing this gap will be essential for improving plant resilience and ensuring sustainable grapevine production under changing environmental conditions.

In addition to post-translational regulation, another emerging but still underexplored aspect of grapevine proteomics is the characterization of vascular (xylem-associated) proteomes. Although a small number of studies have identified proteins involved in cell wall remodeling, defense, and long-distance signaling within xylem sap, comprehensive and systematic analyses remain scarce. Given the central role of vascular tissues in water transport, nutrient distribution, and systemic signaling—particularly during pathogen infection and abiotic stress—future research targeting vascular proteomes is expected to provide important insights into whole-plant integration of stress responses.

Beyond specific stress-related responses, future directions in grapevine proteomics should also be considered in the context of large-scale proteome mapping initiatives. Major international efforts have aimed to systematically characterize complete proteomes, such as the Human Proteome Project, whose goal is to experimentally identify and validate all proteins encoded by the genome. In plant systems, a comparable milestone has been achieved through the development of mass spectrometry–based proteome atlases. For example, a comprehensive draft of the *Arabidopsis thaliana* proteome has been generated, integrating transcriptomic, proteomic, and phosphoproteomic data across multiple tissues and developmental stages, providing quantitative evidence for more than 18,000 proteins and tens of thousands of phosphorylation sites [173]. In contrast, such systematic, organism-wide proteomic resources are still lacking for *Vitis*. The development of comprehensive grapevine proteome atlases, including spatially and temporally resolved datasets, represents an important future goal. Establishing such resources would enable deeper insights into protein function, isoform diversity, and regulatory mechanisms, and would significantly advance the application of proteomics in viticulture and grapevine biology.

Ultimately, the continued advancement of proteomic technologies and their integration with multi-omics approaches will provide deeper insight into the molecular architecture of grapevine biology, facilitating the translation of complex biological knowledge into sustainable and resilient grapevine production systems.

## Figures and Tables

**Table 1:** Proteomic Studies on Grapevine under Biotic Stress.

Stress Factor	Proteomic Method	Main Findings (DEPs and Functional Categories)	Reference
*Plasmopara viticola*	LC–MS/MS	Upregulation of cell wall modifiers, PR proteins, and ROS enzymes; early apoplastic signaling and oxidative burst.	[62]
*Plasmopara viticola*	LC–MS/MS	PR proteins, β-1,3-glucanases, and peroxidases induced; reciprocal host–pathogen communication in apoplast.	[63]
*Plasmopara viticola*	Label-free LC–MS/MS	Activation of primary metabolism enzymes with reduced HR proteins; metabolic maintenance with durable resistance.	[64]
*Plasmopara viticola*	2-DE & LC–MS/MS	Downregulation of photosynthetic proteins and increased PR-10; photosynthesis suppression in susceptible cultivar.	[65]
*Plasmopara viticola*	2-DE & MALDI-TOF/TOF	Induction of PAL, peroxidases, and stilbene enzymes; biocontrol-induced secondary defense activation.	[66]
*Plasmopara viticola*	2-DE & MALDI-TOF/TOF	Decrease in RuBisCO, CAB, and ATP synthase; inhibition of carbon metabolism during compatible interaction.	[67]
*Plasmopara viticola*	2-DE & MS	Increase in OPR-like, amino acid, and carbohydrate metabolism enzymes; PR proteins induced under non-protective response.	[68]
*Plasmopara viticola*	LC–MS/MS	Upregulation of cell wall modifiers and ROS enzymes; apoplast identified as hub of early host–pathogen exchange.	[69]
*Plasmopara viticola*	Comparative 2-DE proteomics	Resistant genotype showed increased ROS detox enzymes and HSPs; susceptible genotype showed photosynthesis inhibition.	[70]
*Plasmopara viticola*	Glycoprotein enrichment & LC–MS/MS	Two glycoproteins linked to stomatal regulation; abnormal opening facilitates infection and sporulation.	[71]
*Erysiphe necator*	iTRAQ LC–MS/MS	Downregulation of photosynthetic proteins and induction of PR-10, APX, and CAT; oxidative burst with defense activation.	[72]
*Erysiphe necator*	2D-DIGE & MS	Maintenance of photosynthesis with increased PR proteins and phenylpropanoid enzymes; Mn priming enhanced defense.	[73]
*Esca disease complex*	2-DE & LC–MS/MS	Upregulation of peroxidases, oxidoreductases, and PAL; decreased energy proteins indicate oxidative defense activation.	[74]
*Esca disease complex*	2-DE & nanoLC–MS/MS	Increased HSP70, sHSP, PR-5, and GST; downregulation of ATP synthase and MDH precedes symptom development.	[75]
*Flavescence dorée*	2-DE & LC–MS/MS	Suppression of photosynthetic proteins with induction of PRs, SOD, GST, and HSP70; strong but ineffective defense response.	[76]
*Flavescence dorée*	Proteomics & phosphoproteomics	Year 1: reduced photosynthesis and primary metabolism; Year 2: partial recovery; altered phosphorylation of signaling proteins.	[77]
*Xylella fastidiosa*	LC–MS/MS	Constitutive expression of chitinases, β-1,3-glucanases, and peroxidases; tolerance linked to xylem defense.	[78]
*Xylella fastidiosa*	LC–MS/MS	PR proteins, antioxidant enzymes, and PGIPs upregulated; xylem defense biomarkers linked to PD tolerance.	[79]
*Xylella fastidiosa*	2-DE & LC–MS/MS	Downregulation of expansins and aquaporins; induction of PR proteins and GST indicating vessel degradation.	[80]
*Xylella fastidiosa*	Sequence/structure bioinformatics	Secretory PR proteins, chitinases, and lectins identified; structural stability supports xylem defense.	[81]
*Xylella fastidiosa*	DIA/ML-enhanced proteomics	Detection of low-abundance PR peptides, metallothioneins, and redox enzymes; extended proteome coverage reveals tolerance components.	[82]

**Table 2:** Proteomic Studies on Grapevine Leaves under Abiotic Stress.

Stress Type	Proteomic Method	Main Findings (DEPs and Functional Categories)	Reference
Water deficit	2-DE & LC–MS/MS	Downregulation of photosynthetic proteins; upregulation of ROS detox enzymes (SOD, APX, GST); accumulation of HSPs and dehydrins.	[85]
Water deficit	2-DE & MALDI-TOF/TOF	Reduction in RuBisCO, increase in antioxidant enzymes and LEA proteins; metabolic reprogramming toward energy balance.	[83]
Water deficit	2-DE & LC–MS/MS	Altered carbohydrate metabolism; upregulation of redox proteins and chaperones; partial recovery upon rewatering.	[86]
Water deficit	Shotgun LC–MS/MS	Enhanced glycolysis and TCA enzymes; accumulation of detox proteins and HSPs; decreased photosynthetic activity.	[84]
Water deficit	Label-free LC–MS/MS	Induction of energy metabolism and redox regulation proteins; decline in light-harvesting complexes and Calvin cycle enzymes.	[87]
Salinity	2-DE & LC–MS/MS	Tolerant cultivar maintained photosynthesis and ROS defense; sensitive showed downregulation of photosynthetic enzymes and upregulation of HSPs.	[85]
Salinity	2-DE & MALDI-TOF/TOF	Decrease in photosynthesis proteins; increase in HSPs, LEA, dehydrins; enhanced glycolytic and TCA cycle enzymes.	[88]
Salinity	2-DE & qPCR	Induction of PR-10 and redox proteins (GST, PDI); link between PR proteins and stress signaling.	[89]
Salinity	Label-free LC–MS/MS	Upregulation of antioxidant and secondary metabolism enzymes; increased aquaporins and ABC transporters in grafted plants.	[90]
Heat stress	iTRAQ LC–MS/MS	Upregulation of HSPs and antioxidant enzymes; downregulation of photosynthesis proteins; recovery phase restored metabolism.	[91]
Heat stress	Integrated proteomics & transcriptomics	Induction of HSPs, PP2Cs, lipid metabolism enzymes; ROS detoxification via AsA–GSH cycle.	[92]
Heat stress	Phosphoproteomics & acetylproteomics	Extensive phosphorylation/acetylation of photosynthesis, signaling, and histone proteins; activation of CDPKs and MAPKs.	[93]
High light irradiance	2-DE & MALDI-TOF/TOF	Upregulation of OEE, CAB, FNR, SOD, APX; enhanced carbon assimilation; minor decline in translation proteins.	[94]
Herbicide stress	2-DE & LC–MS/MS	Induction of GST, CAT, APX, PR proteins; inhibition of RuBisCO and ATP synthase; defense-like detoxification response.	[95]
Photoperiod stress	2-DE & MALDI-TOF/TOF	Increase in LEA, dehydrins, PAL, COMT; decrease in photosynthesis proteins; lignin and flavonoid biosynthesis during dormancy.	[96]
ABA signaling	TMT & phosphoproteomics	Downregulation of photosynthesis proteins; induction of NADP-ME, 6PGD; phosphorylation of SnRK2 targets; activation of ABA signaling.	[97]

**Table 3:** Summary of Proteomic Studies on Grapevine Buds.

Proteomic Method	Main Findings (DEPs and Functional Categories)	Reference
Physiological and biochemical assays with proteomic markers	Short photoperiod-induced dormancy in *Vitis* genotypes; decreased respiration and photosynthesis; increased cold hardiness; downregulation of Calvin cycle enzymes and metabolic slowdown.	[98]
2-DE & immunoblotting of tubulin isoforms	Dynamic reorganization of cytoskeletal proteins during bud ontogenesis; differential expression of α- and β-tubulin and actin; cytoskeletal remodeling associated with dormancy establishment.	[99]
LC–MS/MS-based label-free quantitative proteomics	Seasonal proteome variation throughout the dormancy cycle; accumulation of dehydrins, LEA proteins, and HSPs during deep dormancy; reactivation of energy metabolism and protein translation upon release.	[100]
Label-free LC–MS/MS	Hydrogen cyanamide (HC)-induced dormancy release; induction of GST, peroxiredoxin, hexokinase, sucrose synthase; activation of ABA and auxin signaling; enhanced protein folding and ROS detoxification.	[101]
iTRAQ-based quantitative proteomics and transcriptomics integration	HC-triggered reprogramming of redox and hormonal pathways; upregulation of HSP70/90, MAPKs, and SnRK2; stimulation of carbohydrate metabolism and energy generation during bud break.	[102]
Label-free LC–MS/MS quantitative proteomics	Differentially expressed proteins across dormancy stages; enrichment of antioxidant and stress-response proteins during endodormancy; upregulation of glycolytic and translational proteins in ecodormancy; key redox regulators (SOD, APX) identified.	[103]

**Table 4:** Summary of Proteomic Studies on Grapevine Roots under Abiotic and Nutritional Stress.

Stress Type	Proteomic Method	Main Findings (DEPs and Functional Categories)	Reference
Drought stress	LC–MS/MS proteomics and metabolomic integration	Drought-tolerant rootstock M4 maintained higher levels of antioxidant enzymes (SOD, APX, GST), chaperones (HSP70, HSP90), and LEA proteins. Downregulation of carbon metabolism enzymes (phosphoglycerate kinase, malate dehydrogenase) occurred mainly in the sensitive 101.14 genotype. Proline and polyamine biosynthesis supported osmotic adjustment and redox balance.	[104]
Salinity stress	LC–MS/MS label-free quantitative proteomics combined with metabolomics	Salt exposure induced accumulation of ROS-detoxifying enzymes (peroxiredoxins, catalase, GR) and phenylpropanoid pathway enzymes (CAD, OMT) linked to lignin biosynthesis. Tolerant M4 maintained energy metabolism (ATP synthase, glycolytic and TCA enzymes), while sensitive 101.14 accumulated proteasome-related proteins indicating oxidative damage.	[105]
Cold stress	Label-free quantitative LC–MS/MS proteomics	Cold-tolerant *V. riparia × V. labrusca* roots accumulated dehydrins, SOD, APX, and sucrose–raffinose pathway enzymes for membrane stabilization. Increased levels of 14-3-3 proteins, MAPK-like kinases, and Ca^2+^-binding proteins reflected active signaling and stress adaptation, while Cabernet Sauvignon showed reduced energy metabolism and degradation products.	[107]
Nitrate availability	Label-free LC–MS/MS proteomics	Nitrate deprivation downregulated nitrogen assimilation enzymes (nitrate reductase, glutamine synthetase, glutamate synthase) and enhanced stress-related proteins (SOD, APX, catalase). Adequate nitrate promoted amino acid biosynthesis, energy metabolism, and cell wall remodeling. Proteomic reprogramming indicated tight redox and C–N balance regulation.	[106]

**Table 5:** Proteomic Studies in Grapevine Cell Cultures.

Stress	Proteomic Method	Main Findings (DEPs and Functional Categories)	Reference
Embryogenic vs. non-embryogenic callus	2-DE, MALDI-TOF MS	Upregulation of HSP70, HSP90, SOD, PR proteins, GAPDH, and enolase in embryogenic callus; stress-related and metabolic remodeling associated with embryogenic competence.	[108]
Agrobacterium tumefaciens-mediated transformation callus	2-DE, LC–MS/MS	Altered abundance of ascorbate peroxidase, catalase, 14-3-3 proteins, kinases, and chaperonins; activation of oxidative stress and defense responses.	[109]
Botrytis cinerea infection cell cultures	Label-free LC–MS/MS quantitative proteomics	Induction of PR proteins, protease inhibitors, ROS detoxification enzymes, and amino acid metabolism proteins; activation of defense-related pathways.	[114]
Elicitor treatment	2-DE, LC–MS/MS	Secretion of β-1,3-glucanases, chitinases, PR-5 thaumatin-like proteins, and peroxidases; apoplastic defense activation.	[110]
Methyl-β-cyclodextrin and methyl jasmonate elicitation	2-DE, DIGE, LC–MS/MS	Increased stilbene synthase, PAL, peroxidases, and SOD; activation of phenylpropanoid and oxidative metabolism leading to resveratrol accumulation.	[111]
Developmental model in berry-derived cell suspensions	2-DE, LC–MS/MS	Altered expression of cell wall, lipid, and secondary metabolism proteins reflecting berry ripening-associated processes.	[116]
Jasmonate and Na-orthovanadate elicitation	2-DE, MALDI-TOF MS	Enhanced stilbene synthase, PAL, and oxidative enzymes; stimulation of resveratrol biosynthesis through jasmonate signaling.	[113]
Chitosan treatment	2-DE, MALDI-TOF MS	Induction of defense-related and signaling proteins; redistribution of stilbenes and altered oxidative metabolism.	[112]
Thermal stresses	iTRAQ quantitative proteomics	Modulation of sugar metabolism enzymes, phenylpropanoid pathway proteins, thioredoxin, and GST; metabolic flexibility and antioxidant defense under temperature stress.	[115]
Somatic embryogenesis	2-DE, MALDI-TOF MS	Differential abundance of LTPs, peroxidases, chaperones, and energy metabolism enzymes linked to embryogenic development.	[117]

**Table 6:** Proteomic studies on grape berry ripening.

Proteomic Method	Main Findings (DEPs and Functional Categories)	Reference
2-DE, MALDI-TOF	Downregulation of photosynthetic proteins (RuBisCO, OEE, ATP synthase); upregulation of stress and defense-related proteins during late ripening.	[121]
2D-DIGE, MS/MS	Enhanced glycolytic enzymes (enolase, GAPDH) and TCA cycle proteins (malate dehydrogenase, citrate synthase); decreased organic acid metabolism enzymes (PEP carboxylase, malic enzyme).	[122]
iTRAQ, LC–MS/MS	Induction of glycolytic, mitochondrial, and vacuolar transport proteins; upregulation of ROS scavengers (ascorbate peroxidase, catalase).	[123]
iTRAQ, LC–MS/MS	Increase in vacuolar H^+^-ATPase subunits, TIPs, ABC transporters; activation of vesicle trafficking and stress-related proteins (HSP70, HSP90).	[124]
LC–MS/MS integrated with metabolomics	Upregulation of phenylpropanoid and flavonoid enzymes (PAL, CHS, F3H, STS); correlation with metabolite accumulation during veraison.	[125]
iTRAQ, network analysis	Integration of proteome and metabolome reveals central regulatory nodes connecting sugar metabolism with secondary biosynthesis.	[126]
Label-free LC–MS/MS	Identification of vacuolar and tonoplast transporters; enhanced energy metabolism and secondary metabolite transport during late ripening.	[128]
2-DE, LC–MS/MS	Activation of defense-related and PR proteins (chitinases, GSTs, peroxidases) in Pinot Noir skins; link between ripening and stress priming.	[129]
LC–MS/MS	Induction of amino acid metabolism enzymes (SAMS, aspartate aminotransferase), LOX pathway proteins, and aroma compound biosynthesis.	[130]
LC–MS/MS	Upregulation of ethylene biosynthesis proteins (ACO, SAMS) and lipid transfer proteins; association with volatile production.	[131]
iTRAQ, LC–MS/MS	Stage-specific regulation of glycolytic and amino acid metabolism enzymes; strong accumulation of stress-related proteins during late ripening.	[132]
2-DE, LC–MS/MS	Differential activation of anthocyanin biosynthesis enzymes (CHS, F3H, UFGT) between red and white cultivars.	[127]
LC–MS/MS, metabolomics	Upregulation of HSPs, redox enzymes, and phenylpropanoid proteins in warm-climate Syrah and Cabernet Sauvignon; heat adaptation.	[133]
EST-based database, iTRAQ	Expanded protein annotation coverage; improved identification of low-abundance regulatory and membrane-associated proteins.	[134]

**Table 7:** Proteomic studies on grape berry under abiotic and biotic stress.

Stress Type	Proteomic Method	Main Findings (DEPs and Functional Categories)	Reference
Water deficit	2-DE and LC–MS/MS integrated with metabolomics	Approximately 7% of proteins changed in abundance. Upregulation of PR proteins, proteasome subunits, antioxidant enzymes, and flavonoid biosynthesis enzymes in skins; upregulation of primary metabolism enzymes and glutamate decarboxylase in pulp, reflecting osmotic and oxidative adjustment.	[137]
High temperature	Label-free quantitative LC–MS/MS and metabolomics	Out of 2279 identified proteins, 592 were differentially expressed. High temperature suppressed photosynthetic and glycolytic enzymes, while inducing antioxidant proteins, chaperones, and heat shock–related proteins. Highlights post-transcriptional regulation in thermal adaptation.	[138]
Hormonal (cytokinin) treatment	LC–MS/MS with metabolomic integration	CPPU treatment enhanced glycolytic and TCA cycle enzymes, leading to increased glucose, fructose, and sucrose levels. Demonstrates cytokinin-driven reprogramming of sugar and organic acid metabolism affecting berry growth and ripening.	[139]
Light exposure	Shotgun proteomics (LC–MS/MS)	Light exposure stimulated photosystem II and electron transport proteins while suppressing Calvin cycle enzymes. Induction of antioxidative enzymes suggests a shift toward photoprotection over carbon assimilation.	[140]
Multiple abiotic stresses (heat, water deficit, UV)	Integrative omics (proteomics, transcriptomics, metabolomics)	Combined stresses induced accumulation of anthocyanins, flavonols, and stilbenes, and increased stress-related proteins involved in ROS detoxification and secondary metabolism. Highlights metabolic plasticity and defense cross-talk under environmental stress.	[141]
*Esca proper* (fungal trunk disease)	2-DE + MS (MALDI-TOF/TOF)	Identified 13 DEPs in berry skins; upregulated PR-5 (thaumatin-like), PR-10, polyphenol oxidase, sHSPs; changes in ATP synthase, inorganic pyrophosphatase, aldehyde dehydrogenase, and cysteine synthase; strong activation of oxidative stress and redox pathways affecting amino acid and sulfur metabolism.	[142]
Viral infection (GLRaV-1, GVA, RSPaV)	2-DE + MS-based proteomics integrated with metabolite and agronomic data	Virus-infected Nebbiolo berries showed increased titratable acidity, resveratrol, and induction of oxidative stress enzymes (polyphenol oxidase); altered cytoskeleton- and trafficking-related proteins (α-tubulin, fimbrin, Rab11 GTPase); modulation of phenylpropanoid metabolism and cell structure.	[143]
*Botrytis cinerea* (noble rot)	2-DE + MS (host and pathogen proteins)	Detected both grape and fungal proteins; pathogen markers include peroxiredoxin-5, malate dehydrogenase, ATP synthase α/β, acid protease, and Woronin body protein; host response characterized by changes in antioxidant and phenolic metabolism.	[144]
*Lobesia botrana* infestation	PTM-proteomics (LC–MS/MS with phospho-, glyco-, and acetyl-enrichment)	Quantified 899 proteins; identified >100 phosphorylation sites, 323 N-glycosylation, and 138 lysine acetylation sites; regulation of membrane trafficking, cell wall metabolism, ROS detoxification, and signal transduction; PTMs fine-tune metabolic and structural defense layers.	[145]

**Table 8:** Wine Proteomics.

Proteomic Method	Main Findings (Protein Composition and Function)	Reference
Nano-HPLC–MS/MS (shotgun profiling)	Detected >100 soluble grape-derived proteins; dominance of PR families (thaumatin-like proteins, chitinases), with LTPs, PGIPs, peroxidases; composition varies with cultivar and processing.	[160]
2-DE; MS identification	Defined the major heat-stable proteins persisting in Chardonnay wines; PR proteins (TLPs, chitinases) predominate and explain thermal haze susceptibility.	[161]
2-DE; LC–MS/MS (comparative red wine proteome)	Red wines show lower soluble PR-protein abundance; presence of peroxidases and peptide fragments; strong protein–phenolic interactions shaping solubility and mouthfeel.	[168]
2-DE map; MALDI/ESI–MS	Established a reference map for red wine proteins and peptides; evidence of tannin-binding and oxidative modifications influencing stability.	[167]
LC–MS platforms	State-of-the-art overview; emphasized top-down/peptidomics for intact PR-protein characterization and called for standardization of sample prep and reporting.	[162]
LC–MS/MS; label-free quantification (white wine)	Comprehensive profiling of Silvaner wine (~100 proteins); PR-5/PR-3 most abundant; minor LTPs and enzymes detected depending on vinification.	[169]
Quantitative LC–MS/MS (process comparison)	Pre-fermentation oxygenation and skin contact modulate protein abundance: oxygenation decreases soluble PR proteins; skin contact enriches cell wall–derived proteins/peptides.	[170]
SCX + HIC (native purification); structural downstream use	Two-step non-denaturing purification of PR proteins from wine enabling high-purity isolation for crystallography and functional assays.	[165]
Targeted protein assays; thermal haze tests; LC–MS	Roles in haze formation clarified: chitinase is primary driver of heat instability in white wines; TLPs contribute context-dependently; sulfate enhances aggregation.	[163]
X-ray crystallography; MS support	Resolved structures of haze-forming TLP isoforms; loop flexibility and surface features explain differences in thermal aggregation propensity.	[164]
Protein extraction workflows; biochemical characterization	Optimized extraction of PR proteins from wine; characterized pI, glycosylation and heat-denaturation/aggregation behavior relevant to stability control.	[171]
Comparative LC–MS/MS (varietal/process context)	Quantified chitinases and TLPs in Sauvignon Blanc and Chardonnay; localized origin to skins/pulp; linked abundance to processing and stability outcomes.	[172]
Top-down peptidomics (intact peptide mapping)	Identified intact peptides and cleavage sites from PR proteins in finished wines; partial proteolysis at exposed loops during storage/aging.	[166]

## Data Availability

No new data were created or analyzed in this study. Data sharing is not applicable to this article.

## Abbreviations

The following abbreviations are used in this manuscript:

DEP	Differentially expressed proteins
FAO	Food and Agriculture Organization
HSP70/HSP90	Heat shock proteins 70/90
hpi	Hours post-infection
IPG	Immobilized pH gradient
LC	Liquid chromatography
LC–MS/MS	Liquid chromatography–tandem mass spectrometry
MALDI(-TOF/TOF)	Matrix-assisted laser desorption/ionization (time-of-flight)
MS	Mass spectrometry
OPR	12-oxophytodienoate reductase
PAL	Phenylalanine ammonia-lyase
PAGE	Polyacrylamide gel electrophoresis
PER	Peroxidase
PPO	Polyphenol oxidase
PR proteins (PR-2, PR-3, PR-4, PR-5, PR-10)	Pathogenesis-related proteins
PTMs	Post-translational modifications
ROS	Reactive oxygen species
RuBisCO	Ribulose-1,5-bisphosphate carboxylase/oxygenase
